# Twenty‐four–hour normothermic perfusion of discarded human kidneys with urine recirculation

**DOI:** 10.1111/ajt.14932

**Published:** 2018-06-20

**Authors:** Annemarie Weissenbacher, Letizia Lo Faro, Olga Boubriak, Maria F. Soares, Ian S. Roberts, James P. Hunter, Daniel Voyce, Nikolay Mikov, Andrew Cook, Rutger J. Ploeg, Constantin C. Coussios, Peter J. Friend

**Affiliations:** ^1^ Oxford Transplant Centre Nuffield Department of Surgical Sciences University of Oxford Oxford UK; ^2^ Institute of Biomedical Engineering University of Oxford Oxford UK; ^3^ Department of Cellular Pathology Oxford University Hospitals NHS Foundation Trust John Radcliffe Hospital Oxford UK; ^4^ OrganOx Limited Oxford Science Park Oxford UK

**Keywords:** clinical research/practice, kidney (native) function/dysfunction, kidney transplantation/nephrology, organ perfusion and preservation, translational research/science

## Abstract

Transportable normothermic kidney perfusion for 24 hours or longer could enable viability assessment of marginal grafts, increased organ use, and improved transplant logistics. Eleven clinically declined kidneys were perfused normothermically, with 6 being from donors after brain death (median cold ischemia time 33 ± 36.9 hours) and 5 being from donors after circulatory death (36.2 ± 38.3 hours). Three kidneys were perfused using Ringer’s lactate to replace excreted urine volume, and 8 kidneys were perfused using urine recirculation to maintain perfusate volume without fluid replenishment. In all cases, normothermic perfusion either maintained or slightly improved the histopathologically assessed tubular condition, and there was effective urine production in kidneys from both donors after brain death and donors after circulatory death (2367 ± 1798 mL vs 744.4 ± 198.4 mL, respectively; *P* = .44). Biomarkers, neutrophil gelatinase–associated lipocalin, and kidney injury molecule‐1 were successfully detected and quantified in the perfusate. All kidneys with urine recirculation were readily perfused for 24 hours (n = 8) and exhibited physiological perfusate sodium levels (140.7 ± 1.2 mmol/L), while kidneys without urine recirculation (n = 3) achieved a reduced normothermic perfusion time of 7.7 ± 1.5 hours and significantly higher perfusate sodium levels (159.6 ± 4.63 mmol/:, *P* < .01). Normothermic machine perfusion of human kidneys for 24 hours appears to be feasible, and urine recirculation was found to facilitate the maintenance of perfusate volume and homeostasis.

## Abstract 2

Normothermic machine perfusion of human kidneys for 24 hours is feasible, and urine recirculation facilitates maintenance of perfusate volume and homeostasis.

**Abbreviations**
CIT: cold ischemia time
DBD: donor after brain death
DCD: donor after circulatory death
HMP: hypothermic machine perfusion
IRR: intrarenal resistance
KIM‐1: kidney injury molecule‐1
NGAL: neutrophil gelatinase–associated lipocalin
NMP: normothermic machine perfusion
SCS: static cold storage
TPN: total parenteral nutrition
WIT: warm ischemia time

## 1 INTRODUCTION

As outcomes of kidney transplant have improved and the incidence of renal failure has increased, the indications and demand for transplant have expanded. Meanwhile, the number of available “ideal” donors, those associated with the best posttransplant outcomes, has declined. The transplantation community has therefore increasingly turned to the use of older and higher‐risk donor organs, those that would not previously have been considered acceptable for transplant. Unfortunately, many such organs are not transplanted, sometimes because of clear evidence of nonviability but more often due to uncertainty as to the quality of the organ. In the past years, there has been a resurgence of interest in hypothermic machine perfusion (HMP). There is evidence that this form of preservation is superior to static cold storage (SCS), especially in the context of higher‐risk and extended criteria donor organs.^1^, ^2^ Recently, the addition of oxygen delivered in some experimental perfusion systems has been shown to further improve outcomes in HMP.^3^, ^4^ To date, however, the “gold standard” in solid organ preservation in many centers remains static cold storage. Although HMP studies have shown a decrease in delayed graft function and better survival,^1^, ^2^ improved use of marginal kidneys, including from donors after cardiac death (DCDs), has remained elusive.

Normothermic machine perfusion (NMP) provides several potential advantages over both SCS and HMP by enabling normal cellular metabolism with recovery of cellular energetic status, allowing repair of reversible injury, and facilitating functional testing of the organ before transplant through the measurement of multiple perfusion and biochemical parameters during preservation.

The direct evidence that kidney preservation by NMP is superior to SCS is based on an increasing number of clinical and experimental studies from Hosgood/Nicholson and Selzner.^5^, ^6^, ^7^, ^8^ The preclinical studies by Hosgood/Nicholson et al demonstrated the potential for delivering therapy to the organ during normothermic perfusion,^9^, ^10^ and the authors were first to demonstrate that a short, 2‐ or 1‐hour period of normothermic perfusion before transplant after SCS yielded significant improvements in metabolic function and reduced tubular injury compared with SCS alone.^11^, ^12^ These findings were rapidly translated into human data in a series of seminal studies that showed that a 60‐minute period of normothermic perfusion after SCS made it possible to transplant marginal kidneys more reliably and with greatly improved immediate graft function compared with SCS alone.^13^, ^14^, ^15^, ^16^

All clinical studies to date have targeted short‐term durations of perfusion (1‐2 hours), with the intention of recovering cellular energetics before reperfusion in the recipient.^5^, ^13^, ^16^ Brasile et al described successful perfusion of isolated canine and human kidneys ex vivo hypothermically at 32ºC for 48 hours.^17^ However, recent work by Selzner et al in a porcine model of kidney transplant demonstrated that the use of longer, 16‐hour periods of NMP after SCS is superior to both SCS alone and short‐duration NMP, in terms of both tubular injury and organ function posttransplant.^18^, ^19^ Recent phase 1 and phase 3 studies in human liver transplant have further evidenced the superiority of NMP over SCS for both conventional and extended criteria donor livers,^20^, ^21^ but to date there are no published studies of prolonged NMP perfusion of human kidneys.

The aim of the present study is to evaluate for the first time the feasibility of longer‐term transportable NMP in human kidneys for periods of up to 24 hours. Urine recirculation has been used to facilitate maintenance of a constant perfusate volume without additional fluid replenishment during that time period. The purpose of this study was to establish not whether urine recirculation is superior but whether it is feasible.

## 2 MATERIAL AND METHODS

Thirteen human kidney grafts were included in this study. All organs were retrieved for the purpose of transplant but discarded during postretrieval assessment at the donor hospital or at the transplant center. After being sent to Oxford, perfusions were performed at the Institute of Biomedical Engineering, University of Oxford. Hemodynamic and biochemical perfusion parameters were analyzed. Perfusate samples and biopsy samples were obtained at regular time points during perfusion. The study was evaluated and approved by the National Ethics Review Committee of the United Kingdom (REC reference 12/EE/0273 IRAS project ID 106793).

### 2.1 Preparation of kidney grafts

Kidneys were retrieved with the intention of clinical transplant during standard multiorgan recovery in donor hospitals in the United Kingdom. Organs were preserved with either Marshall hypertonic citrate solution (Soltran Baxter Healthcare, Thetford, UK) or UW solution (Belzer UW CSS, Bridge to Life, Columbia, SC), packed, and shipped according to standard clinical practice. On arrival at the research laboratory, the kidneys were immediately assessed and prepared for connection to the NMP circuit. Back table preparation and priming of the perfusion machine were carried out in parallel. If necessary, accessory arteries were reconstructed during the back table preparation to provide a single inflow. Cannulation of the renal artery and the renal vein was carried out with a 20‐Fr and a 10‐Fr cannula. The ureter was cannulated with a 6‐Fr or an 8‐Fr tube to recirculate the urine. Urine production was continuously monitored by using an inline flow sensor (LD20 Liquid Flow Sensor; Sensirion AG, Stäfa, Switzerland). All cannulas were secured with ligatures; 30‐40 minutes were needed for graft preparation. Back table preparation was performed with kidneys still immersed in ice‐cold preservation solution. Grafts were then flushed with approximately 200 mL of crystalloid Ringer’s lactate at room temperature, before connection to the NMP device.

**Figure 1:**
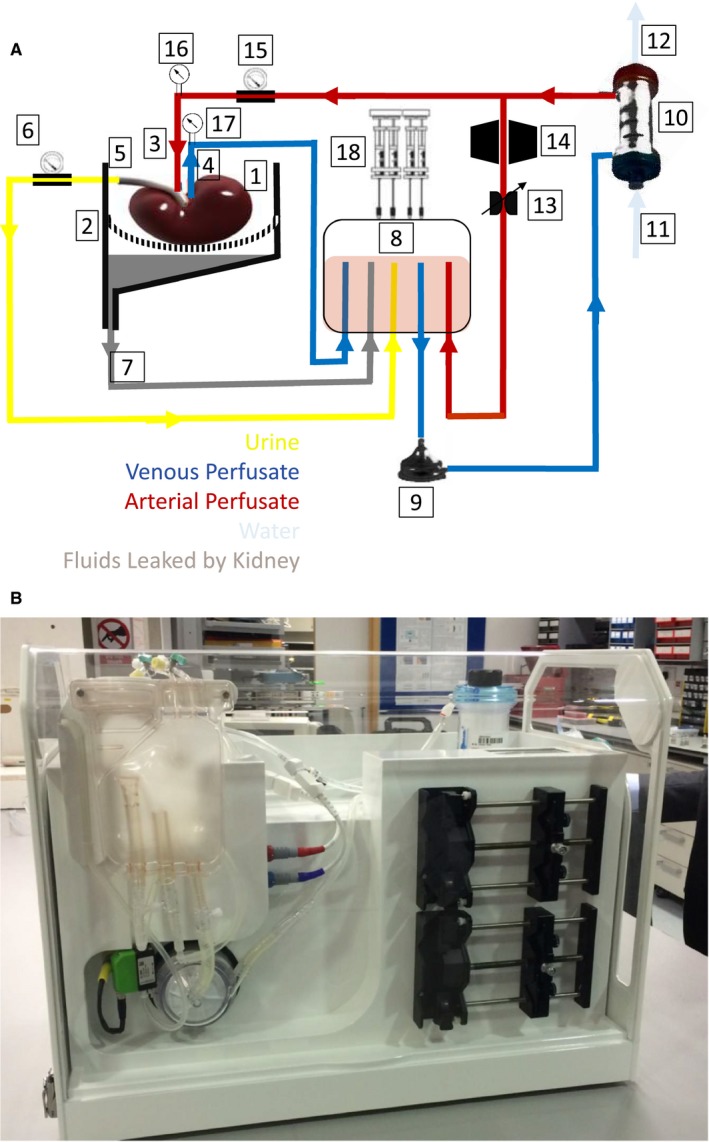
A. Diagram/schematic drawing of the prototype device for long‐term normothermic kidney perfusion: (1) Kidney. (2) Organ container, including perforated kidney sling. (3) Arterial cannula at kidney inlet. (4) Venous cannula at kidney outlet. (5) Ureter outlet duct. (6) Urine flow meter. (7) Duct for recirculation of fluids leaked by the kidney. (8) Soft‐shell reservoir. (9) Perfusion pump (centrifugal pump). (10) Oxygenator & heat exchanger. (11) Heat exchanger water inlet. (12) Heat exchanger water outlet. (13) Proportional pinch valve (capable of both fixed & alternating constriction). (14) In line blood gas analysis sensor capable of measuring temperature, po_2_, pco_2_, pH. (15) Ultrasonic arterial flow meter. (16) Arterial pressure gauge. 17 Venous pressure gauge. (18) Syringe pump. B. Prototype device for long‐term normothermic kidney perfusion (OrganOx Ltd, Oxford, UK) [Color figure can be viewed at wileyonlinelibrary.com]

A diagram and the perfusion device are shown in Figure 1A,B. The system was designed to support kidneys ex vivo for a prolonged preservation period by using perfusion with an oxygenated suspension of packed red blood cells (RBCs) in a colloid, supplemented by nutrients, at normal body temperature. The components of the circuit were sourced from cardiopulmonary bypass suppliers and consisted of a blood reservoir (Capiox venous reservoir, Terumo Medical Corporation, Somerset, NJ), centrifugal blood pump (AFFINITY CP centrifugal blood pump; Medtronic, Minneapolis, MN), membrane oxygenator/heat exchanger Sorin (Lilliput 2 extracorporeal membrane oxygenator, LivaNova PLC, London, UK) and medical‐grade silicone tubing with internal diameters of 1/4 and 3/8 inch. A custom‐built thermoelectric heater and cooler were used to maintain the blood temperature at 37°C. Pressure in the renal artery was measured in‐line with single‐use pressure sensors (PendoTECH sensors; PendoTECH, Princeton, NJ). Hemodynamic control was based on arterial pressure, without any direct adjustment of arterial flow and automatically maintained in the range of 70‐100 mm Hg via continuous adjustment of the centrifugal pump speed between 1300 and 1500 rpm. The flow through the kidney was measured with an external ultrasonic flow sensor (Sonoflow CO.55/080; SonoTec, Halle, Germany). Blood gases were automatically maintained within physiological limits by using a closed‐loop controller, consisting of an oxygen concentrator and air compressor that delivered oxygen flow rates on the order of 10 mL/min and air flow rates on the order of 100 mL/min. Proportional control valves were used to automatically regulate gas flow rates to maintain po_2_ between 10 and 26 kPa and pco_2_ between 2 and 6 kPa.

### 2.2 Management of fluid volume loss due to urine production

The rate of urine production was continuously measured in all kidneys by using a liquid flow sensor (LD20 Liquid Flow Sensor; Sensirion AG). In kidneys without urine recirculation (n = 3), perfusate volume was maintained by immediately replacing every 20‐30 mL of excreted urine with an equivalent volume of Ringer’s lactate, as described by previous investigators.^18^, ^19^ In the remaining kidneys (n = 10), urine was recirculated into the circuit to maintain a constant circulating volume and to avoid the electrolyte imbalance that would result from replacement with crystalloid fluids. The effect of urine recirculation in a normothermic kidney perfusion circuit has not been reported previously.

### 2.3 Perfusion solution

During preparation of the kidney for cannulation, the perfusion circuit was assembled and primed with 1 unit of packed RBCs of the same blood group as the kidney, resuspended in 250 mL of 5% human albumin solution, giving a total perfusate volume of 500 mL. During priming of the circuit, before connection of the kidney, the perfusate was supplemented with bolus doses of cefuroxime (750 mg) and calcium gluconate 10% (10 mL to counteract the calcium binding of citrate). The pH was adjusted through titration with sodium bicarbonate 8.4% (5‐15 mL) to the physiological level of 7.3. No additional sodium bicarbonate was given at any point during perfusion after kidney connection. After perfusion start, 10 mL of 10% mannitol was added. During the perfusion, epoprostenol sodium (Flolan 0.5 mg; GSK, Brentford, UK) was infused at a constant rate of 4 μg/h, to optimize the microcirculation. For glucose control, blood glucose measurements were carried out every 2 hours. A bolus injection of 2.5 mL of a lipid‐free parenteral nutrition solution (total parenteral nutrition [TPN]) (Nutriflex Special; B. Braun AG, Melsungen, Germany; containing 0.24 mg/mL glucose) was administered once blood glucose levels dropped below 4 mmol/L. The temperature of the perfusate was maintained at 37°C throughout the perfusion period.

### 2.4 Biochemistry

Blood gas analysis was performed by using an in‐line blood gas analyzer (CDI 500, Terumo Medical Coroporation, Somerset, NJ). Glucose measurements were performed off‐line using a hand‐held blood gas analyzer (iSTAT, Abbot Point of Care Inc., Princeton, NJ), which was also used to further confirm Po2 and Pco2, and pH arterial and venous measurements where needed.

### 2.5 Potential biomarkers: neutrophil gelatinase–associated lipocalin and kidney injury molecule‐1 measurements in the perfusate

Perfusate samples were collected at 5 time points (at 1, 6, 12, 18, and 24 hours) during NMP and processed via centrifugation at 4000 rpm for 15 minutes at 4°C. The supernatant was aliquoted and snap‐frozen. Neutrophil gelatinase–associated lipocalin (NGAL) and kidney injury molecule‐1 (KIM‐1) levels in the perfusate samples were measured by using a quantitative sandwich enzyme immunoassay technique with NGAL and KIM‐1 Quantikine ELISA kits (R&D Systems Minneapolis, MN) according to the manufacturer's instructions. Briefly, after preparation of the reagents, the assay buffer, standards (recombinant human NGAL or KIM‐1), positive controls, and perfusate samples were added to the wells of a 96‐well plate coated with a monoclonal antibody specific for human NGAL or KIM‐1, respectively. After incubation (2 hours at 4°C for NGAL and 2 hours at room temperature for KIM‐1 according to manufacturer's instructions), wells were emptied via aspiration and washed 4 times with the wash buffer provided. After the last wash, secondary antibodies specific for NGAL and KIM‐1, conjugated to horseradish peroxidase, were added to each well. Plates were sealed and incubated (2 hours at 4°C for NGAL and 2 hours at room temperature for KIM‐1). After incubation, each well was emptied and again washed 4 times. Horseradish peroxidase substrates (hydrogen peroxide and the chromogen tetramethylbenzidine) were added to each well and incubated for 30 minutes at room temperature while protected from light. After 30 minutes, the reaction was stopped by the addition of sulfuric acid and the plates were read at 450 nm (with wavelength correction at 540 nm) for NGAL and KIM‐1, in a microplate reader (BMG Omega plate reader, BMG Labtech, Ortenberg, Germany). Biomarker levels in perfusate samples were extrapolated from the standard curves.

### 2.6 Histology

Core needle biopsy specimens were fixed in Millonig solution and processed for paraffin embedding. The 3‐μm‐thick sections were stained with hematoxylin and eosin. Light microscopy observations were carried out with the use of a Nikon Eclipse 50i microscope (Nikon Corporation, Tokyo, Japan). Histological assessment was based on modified Remuzzi scores^22^ and on the following parameters: number of glomeruli, percentage of globally sclerosed glomeruli, percentage of chronic damage (interstitial fibrosis and tubular atrophy in the cortex), arteriolar hyalinosis (0, absent; 1, present), intimal elastosis (0, absent; 1, less than or equal to the thickness of the media; 2, greater than the thickness of the media), and presence of acute tubular injury (0, absent; 1, loss of brush borders/vacuolation of tubular epithelial cells; 2, cell detachment/cellular casts; 3, coagulation necrosis). Immunohistochemistry for KIM‐1 was performed (human TIM‐1/KIM‐1/HAVCR antibody, monoclonal mouse IgG2B clone #219211; R&D Systems) after heat‐induced antigen retrieval, incubation with primary and secondary antibodies, staining with diaminobenzidine, and hematoxylin counterstaining. Positivity was characterized by brown staining in the apical borders in epithelial cells from proximal tubuli or from the outer stripe of the renal medulla. The percentage of stained tubules and their location were noted.

The analyzing pathologist was blinded for the duration of cold ischemia time (CIT), the timing of the kidney biopsy, and the type of perfusion (with or without urine recirculation).

### 2.7 Statistical analysis

For statistical evaluation of the data (excluding ELISA), unpaired *t* tests (parametric) and Mann‐Whitney tests (nonparametric) were performed using GraphPad Prism 7. A *P*‐value of <.05 was considered significant.

## 3 RESULTS

### 3.1 Donor characteristics

**Figure 2:**
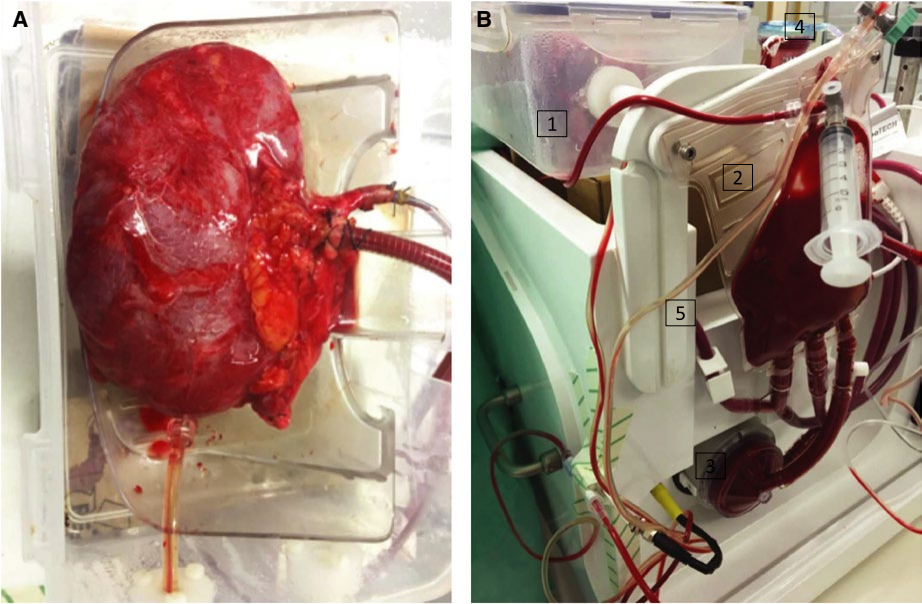
A. Kidney 3 after 24 hours of normothermic perfusion. B. Prototype device in operation mode. (1) Organ container with kidney. (2) Soft‐shell reservoir. (3) Perfusion pump (centrifugal pump). (4) Oxygenator & heat exchanger. (5) Urine recirculation line [Color figure can be viewed at wileyonlinelibrary.com]

Table 1 summarizes organ retrieval characteristics and the reasons the 11 included kidneys had been declined for clinical use. For logistical reasons, discarded human kidneys offered for research typically have a longer CIT than do most of the transplanted kidneys. Figure 2A shows kidney 3 after 24 hours of normothermic perfusion; Figure 2B displays the perfusion device in operation mode. Kidneys 4 and 8 had an extraordinarily long CIT of >100 hours for logistical reasons. Kidney 3 had the shortest CIT as the organ was declined at the Oxford Transplant Centre due to the presence of a renal neoplasm (removed). Donor demographics are summarized in Table 2. Four DBD and 4 DCD kidneys were perfused for 24 hours with urine recirculation. Two (1 DBD, kidney 12; 1 DCD, kidney 13) experienced a device malfunction resulting in either no vasodilator infusion or reduced nutrition: these 2 kidneys are thus presented separately in Supplementary Information. An additional 3 kidneys (2 DCD and 1 DBD) were perfused without urine recirculation, which led to a significant shorter time on the perfusion device compared with the 10 kidneys with urine recirculation (*P* < .0001). All demographic parameters were similar in the 2 groups. Volume loss due to urine production was replenished 1:1 with Ringer’s lactate solution. Reasons for termination of the kidney perfusions were (i) arterial flow ≤50 mL/min, (ii) pH <7 or >7.7 measured at a pco_2_ level of 5, and/or (iii) sudden cessation of urine production.

**Table 1:** Organ procurement parameters and reasons for discard of individual kidneys

	Age, y	Sex	BMI, kg/m^2^	Donor type	WIT, min	CIT, h + min	Hypertension	Reason for discard
Kidney 1	61	Male	24	DBD	N/A	19 + 4	No	Esophageal cancer
Kidney 2	73	Male	35.8	DBD	N/A	20 + 39	Yes	Patchy perfusion
Kidney 3	51	Female	22	DCD	18	11 + 23	No	Renal neoplasia
Kidney 4	44	Female	26.4	DCD	33	104 + 3	No	Patchy perfusion cortical necrosis
Kidney 5	49	Male	25.7	DCD	12	25 + 18	No	Patchy perfusion
Kidney 6	68	Male	23.2	DBD	N/A	108 + 5	No	Patchy perfusion
Kidney 7	47	Female	39.1	DBD	N/A	12 + 41	No	Vascular damage, patchy perfusion
Kidney 8	62	Female	23.5	DCD	9	19 + 52	No	Suspicion of cancer
Kidney 9	74	Female	24.8	DCD	11	22	Yes	Lesion on partner kidney (monomorphic cell infiltration)
Kidney 10	78	Female	25.4	DBD	N/A	18 + 22	Yes	Vascular damage
Kidney 11	71	Female	29.1	DBD	N/A	21 + 4	No	Organ size

DCD, donation after circulatory death; DBD, donation after brain death; CIT, cold ischemia time; WIT, warm ischemia time; N/A, not applicable.

**Table 2:** Donor characteristics

	DBD (n = 6)	DCD (n = 5)	*P*‐value
Donor age, y (median, min‐max)	69.5 (47‐78)	51 (44‐74)	.17
Donor BMI, kg/m^2^	29.4 ± 6.6	24.5 ± 1.8	.14
Serum creatinine at admission, μmol/L	99 ± 41	82 ± 27	.48
Serum creatinine at retrieval, μmol/L	93 ± 35.1	47.5 ± 15.6	.04
Serum urea at admission, mmol/L	6.2 ± 2.5	4.1 ± 0.7	.22
Serum urea at retrieval, mmol/L	6.7 ± 3.3	7.7 ± 6.4	.75
Urine output last hour, mL	165 ± 142	105 ± 83.7	.43
Urine output last 24 h, mL	34 500 ± 1244	4117 ± 3232	.66
CIT, h	33 ± 36.9	36.2 ± 38.3	.89
WIT, min	N/A	16.2 ± 10	N/A

Unless otherwise indicated, values are mean ± SD.

DBD, donation after brain death; DCD, donation after circulatory death; CVA, cerebro vascular accident; CIT, cold ischemia time; WIT, warm ischemia time; N/A, not applicable.

### 3.2 Hemodynamic and metabolic function parameters

**Figure 3:**
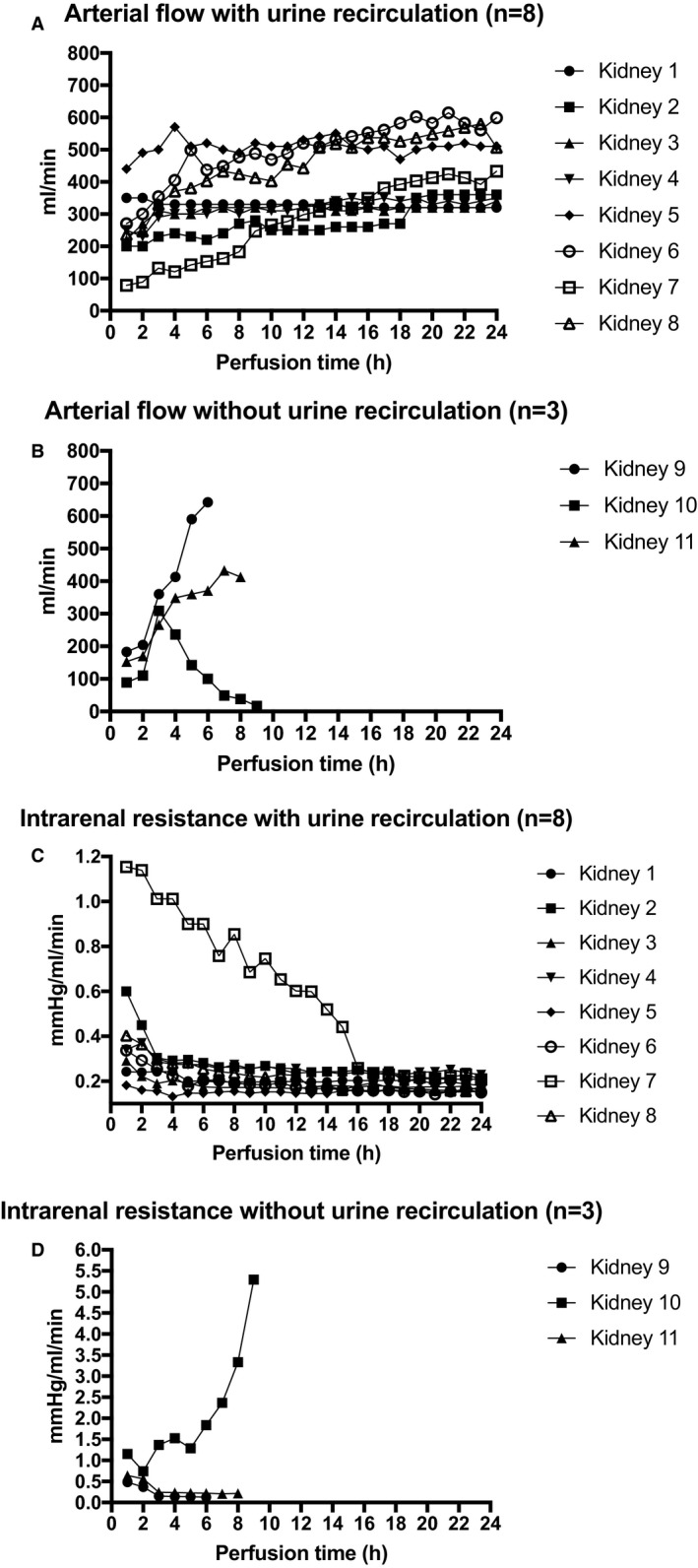
A. Arterial flow values in mL/min per kidney with urine recirculation over time. B. Arterial flow values in mL/min per kidney without urine recirculation over time. C. Intrarenal resistance values in mm Hg/mL/min per kidney with urine recirculation over time. D. Intrarenal resistance values in mm Hg/mL/min per kidney without urine recirculation over time

Perfusion parameters for the overall cohort, with or without urine recirculation, and the mean ± SD values for DBD and DCD kidneys are shown in Table 3. Physiological mean arterial pressures and flows could be achieved in both donor categories in the urine recirculation group and in 2 of the kidneys without urine recirculation. The hemodynamic parameters are shown in Figure 3A,B (arterial flow) and 3C,D (intrarenal resistance).

**Table 3:** Perfusion characteristics

	Kidneys with urine recirculation			Kidneys without urine recirculation			*P*‐value^a^
	Overall (n = 8)	DBD (n = 4)	DCD (n = 4)	Overall (n = 3)	DBD (n = 2)	DCD (n = 1)	
Arterial pressure in mmHg (mean, SD)	77.66 ± 12.93	78.82 ± 13.23	76.5 ± 14.6	90.1 ± 0.63	90.4 ± 0.5	89.5	.14
Arterial flow in mL/min (mean, SD)	372.1 ± 117.5	331.1 ± 129.6	413 ± 104.7	319 ± 198.2	235.5 ± 191.6	486	.59
IRR in mmHg/mL/min (mean, SD)	0.23 ± 0.08	0.26 ± 0.09	0.19 ± 0.04	0.44 ± 0.4	0.58 ± 0.5	0.18	.14
pH (mean, SD)	7.38 ± 0.08	7.32 ± 0.07	7.43 ± 0.03	7.49 ± 0.19	7.43 ± 0.23	7.6	.16
pO_2_ in kPa (mean, SD)	16.3 ± 4.9	17 ± 5.8	15.4 ± 4.8	14.5 ± 0.5	14.3 ± 0.4	15	.57
pCO_2_ in kPa (mean, SD)	5 ± 0.1	5 ± 0.7	5.2 ± 0.4	4.9 ± 0.2	5.1 ± 0.1	4.8	.65
Overall urine output in mL (mean, SD)	1985 ± 3800	3151 ± 5464	819.3 ± 474.3	680 ± 237.5	797.5 ± 173.2	445	.58
Urine output in mL/h (mean, SD)	82.71 ± 158.3	131.3 ± 227.6	34.14 ± 19.8	86.9 ± 14.2	93.3 ± 12.6	74.17	.97
Amount of glucose in mg/perfusion duration (mean, SD)	5.6 ± 2.4	6.8 ± 3	4.4 ± 1	0.85 ± 0.6	1 ± 0.7	0.55	.0093
Time on circuit in h (mean, SD)	24.1 ± 0.2	24.1 ± 0.2	24.2 ± 0.2	7.7 ± 1.5	8.5 ± 0.7	6	<.0001

DBD, donation after brain death; DCD, donation after circulatory death.

a Comparing values for the 8 kidneys with urine recirculation vs. the values for the 3 kidneys without urine recirculation.

#### 3.2.1 Arterial pressure

The arterial pressure was regulated by changing the pump speed with the aim of maintaining a physiological mean arterial pressure between 70 and 100 mm Hg throughout the perfusion. Detailed pressure values per kidney are shown in Table 4.

**Table 4:** Hemodynamic and metabolic function parameters per kidney

	Kidney 1	Kidney 2	Kidney 3	Kidney 4	Kidney 5	Kidney 6
Arterial pressure, mm Hg^a^	67.2 ± 7.2	73.7 ± 14.8	56.5 ± 2.5	81.7 ± 1.5	77.8 ± 1.6	78.9 ± 6.2
Arterial flow, mL/min^a^	327.5 ± 8.5	269.4 ± 57	311.7 ± 23.5	318.3 ± 30.6	509.6 ± 25.2	498.8 ± 92.9
IRR, mL/min/mm Hg^a^	0.21 ± 0.02	0.27 ± 0.09	0.18 ± 0.03	0.26 ± 0.03	0.15 ± 0.01	0.19 ± 0.05
pH^a^	7.34 ± 0.12	7.24 ± 0.04	7.44 ± 0.08	7.45 ± 0.09	7.44 ± 0.06	7.38 ± 0.09
arterial po_2_/kPa, mm Hg^a^	21.2 ± 6.1/159.3 ± 45.8	26.1 ± 12.7/196.2 ± 95.4	26.5 ± 13.5/199 ± 101.5	12.2 ± 2.6/91.7 ± 19.5	13.5 ± 0.7/101.5 ± 5.3	12.5 ± 1.5/94 ± 11.3
venous po_2_/kPa, mm Hg^a^	9.2 ± 3.2/69 ± 24	9.2 ± 2.9/68 ± 21.8	10.1 ± 3/199 ± 101.5	7.2 ± 1.1/54.1 ± 8.3	7.3 ± 1.1/54.9 ± 8.2	7.6 ± 1.4/57.1 ± 10.5
arterial pco_2_/kPa, mm Hg^a^	4.9 ± 1.6/36.8 ± 12	5.9 ± 0.4/44.3 ± 3	5.6 ± 1/42.1 ± 7.5	4.8 ± 1.8/36.1 ± 13.5	5 ± 0.3/37.6 ± 2.3	5.5 ± 2.6/41.4 ± 7.5
Lactate level, mmol/L^a^	17.71 ± 2.7	18.77 ± 2	13.57 ± 4.2	16.14 ± 2.3	10.92 ± 0.9	7.78 ± 2.3
Total glucose, mg^b^	10.8	7.2	4.8	5.28	4.5	4.32
Total urine output, mL/24 h	984	140	1078	264	610	155
Urine recirculation, yes/no	Yes	Yes	Yes	Yes	Yes	Yes
Time on the device (h + min)	24 + 19	24 + 4	24 + 12	24	24 + 19	24

	Kidney 7	Kidney 8	Kidney 9	Kidney 10	Kidney 11
Arterial pressure, mm Hg^a^	91.64 ± 2.3	89.2 ± 2.2	88.9 ± 1.7	92.2 ± 2.8	89.8 ± 0.5
Arterial flow, mL/min^a^	240.7 ± 120.9	468.8 ± 82.8	474.1 ± 149.7	123.5 ± 79.16	339.6 ± 83.4
IRR, mL/min/mm Hg^a^	0.5 ± 0.3	0.19 ± 0.05	0.2 ± 0.09	1.2 ± 1.02	0.3 ± 0.12
pH^a^	7.33 ± 0.1	7.39 ± 0.04	7.66 ± 0.2	7.2 ± 0.1	7.6 ± 0.2
arterial po_2_/kPa, mm Hg^a^	13.5 ± 2.9/101.5 ± 21.8	15.4 ± 3.6/115.8 ± 27	15 ± 3/112.8 ± 22.5	14.6 ± 2/109.8 ± 15	14 ± 3.2/105.3 ± 24
venous po_2_/kPa, mm Hg^a^	6.7 ± 1.3/50.4 ± 9.8	7.1 ± 1.6/53.4 ± 12	7.4 ± 0.9/55.6 ± 6.8	7.8 ± 1.8/58.6 ± 13.5	6.4 ± 1.3/48.1 ± 9.8
arterial pco_2_/kPa, mm Hg^a^	4.5 ± 0.9/33.8 ± 6.8	4.7 ± 0.6/35.3 ± 4.5	4.6 ± 0.6/34.6 ± 4.5	4.8 ± 1.1/36.1 ± 8.3	5 ± 0.7/37.6 ± 5.3
Lactate level, mmol/L^a^	18.84 ± 2.2	9.19 ± 2	9.62 ± 4	14.28 ± 4.5	16.34 ± 2.9
Total glucose, mg^b^	5	3	0.55	1.5	0.5
Total urine output, mL/24 h	11325	1325	445	920	675
Urine recirculation, yes/no	Yes	Yes	No	No	No
Time on the device (h + min)	24 + 5	24	6 + 10	9 + 25	8 + 10

Unless otherwise indicated, values given as mean ± SD.

a Time‐averaged longitudinal mean value compiled from hourly measurements over the course of the perfusion.

b Circulating perfusate volume of 500 mL.

#### 3.2.2 Arterial flow

Arterial flow for each single kidney is shown in Figure 3A,B and Table 4. Overall, the best arterial flow was achieved in kidney 5, a DCD kidney with a CIT of >24 hours and with a mean arterial flow of 509.6 ± 25.2 mL/min. This kidney had a significantly better flow rate during the entire perfusion of 24 hours than all other kidneys (*P* < .0001). In kidneys 4 and 6, kidneys with an extraordinary long CIT of >100 hours (respectively discarded because of poor perfusion and histological signs of cortical necrosis, and because of patchy perfusion^8^), excellent arterial flows were achieved, significantly better than the flow rates in kidney 2 (*P* = .001; both DBD kidneys from elderly donors with CIT <24 hours [discarded due to poor perfusion at retrieval]). In kidney 10 (perfusion with urine replenishment), the arterial flow decreased drastically to 142 mL/min (before 309 mL/min) after 4 hours on the prototype and replenishment of 700 mL of urine with Ringer’s lactate. From hour 7 on, the flow in kidney 10 was <50 mL/min.

#### 3.2.3 Intrarenal resistance

Intrarenal resistance (IRR) was calculated by dividing pressure at a specific time point by the flow at the same time point. The changes of IRR over time are shown in Figure 3C,D and Table 4. As with arterial flow, IRR was lowest in kidney 5 compared with the other perfused kidneys; mean IRR was 0.15 ± 0.01 mm Hg/mL per minute (*P* < .0001). The highest IRR levels could be observed in kidney 10: 1.2 ± 1.02 mm Hg/mL per minute.

#### 3.2.4 Po_2_ and Pco_2_ levels

Mean arterial po_2_ and pco_2_ levels for each kidney are displayed in Table 4. Arterial po_2_ levels were measured by using a Terumo CDI 500 device and i‐STAT CG4+ cartridges. Venous po_2_ levels were measured with i‐STAT CG4+ cartridges only. The differences between arterial and venous oxygenation were significant in the perfused kidneys: artery 20.24 ± 1.17 kPa versus vein 10.7 ± 0.7 kPa, *P* < .0001.

#### 3.2.5 pH levels

**Figure 4:**
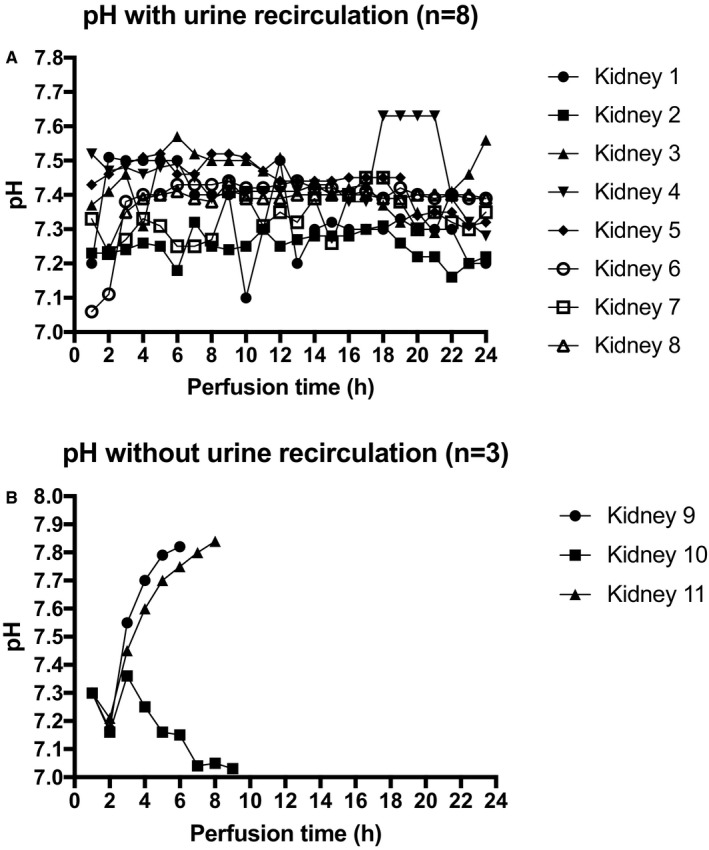
A. pH values per kidney with urine recirculation during 24 hours of normothermic perfusion. B. pH values per kidney without urine recirculation during 24 hours of normothermic perfusion

The perfusate osmolality at perfusion start was 281 ± 5 mOsm/kg. The course of pH for all perfused kidneys is shown in Figure 4A,B and displayed in Table 4. In all perfusions, 10‐15 mL of sodium bicarbonate 8.4% was given to reach a physiological pH, >7.2 at 37°C, before starting perfusion. No further sodium bicarbonate was given at any point during perfusion.

The pH developed interestingly in the 3 kidneys without urine recirculation. Kidney 9 became very alkalotic with a pH >7.7 after 4 hours of perfusion and replenishment of 405 mL with Ringer's lactate. The pco_2_ levels were physiological throughout (4.6 ± 0.6). Urine production stopped after 4 hours 30 minutes; the perfusion was terminated after 6 hours. Kidney 10 developed a slightly acidotic pH (pco_2_ = 4.8 ± 1.1) after hour 6 in combination with a steadily decreasing arterial flow and lack of/stop of urine production from the same time onward. The perfusion was stopped after 9 hours 30 minutes with an arterial flow of 17 mL/min. Kidney 11 had a good arterial flow throughout the 8 hours of normothermic perfusion. The perfusion was stopped as the kidney became very alkalotic with ph >7.8 (pco_2_ = 5 ± 07) and stopped producing urine after 6 hours 30 minutes; 550 mL of urine have been produced/replenished until the end of hour 5 after the start of the perfusion.

#### 3.2.6 Glucose measurements

The perfusions included administration of nutrition throughout the 24‐hour perfusion period. We used lipid‐free TPN solution Nutriflex Special (B. Braun Melsungen AG), which contains glucose (396 g glucose monohydrate; 0.24 mg glucose/mL); overall 5190 kJ (1240 kcal)/L solution was used. The overall amount of glucose given throughout the perfusion is shown in Tables 3 and 4.

Evaluation of glucose consumption in the case of an isolated kidney is not readily achievable, because there is not only glucose consumption but also gluconeogenesis, glycolysis, glucose filtration, and glucose reabsorption taking place at the same time.

#### 3.2.7 Lactate levels during perfusion

The lactate levels for each single kidney are shown in Table 4. The median lactate levels after 1 hour, after 6 hours, and at the end of perfusion (after 24 hours) were 11.99 mmol/L (10.53‐15.72), 15.13 mmol/L (6.6‐20), and 15.41 mmol/L (4.8‐20) in the urine recirculation group. The median lactate levels for the urine replenishment group were 12.84 mmol/L (9.87‐12.9) after 1 hour, 16.27 mmol/L (4.51‐20) after 6 hours, and 17.82 mmol/L (4.51‐19.65) at the end of perfusion. There was no correlation between perfusion parameter, histological results, perfusate sodium, and lactate levels.

#### 3.2.8 Urine production and sodium levels in perfusate and urine

All kidneys produced urine. The average 24‐hour urine production of the 8 kidneys with urine recirculation was 620 mL (140‐11325 mL). Median urine production of kidneys without urine recirculation (n = 3) was 675 mL (445‐920 mL). Kidney 7 was polyuric with very high volumes (>400 mL/h) of urine production until hour 20. After this time, urine production decreased to about 70 mL/h. Urine production is shown in Tables 3 and 4.

Perfusate sodium levels of kidneys with urine recirculation were in a physiological range during the 24 hours of perfusion: 140.7 ± 1.2 mmol/L. Perfusate sodium levels in kidneys without urine recirculation were significantly higher: 159.6 ± 4.63 mmol/L, *P* < .01.

**Figure 5:**
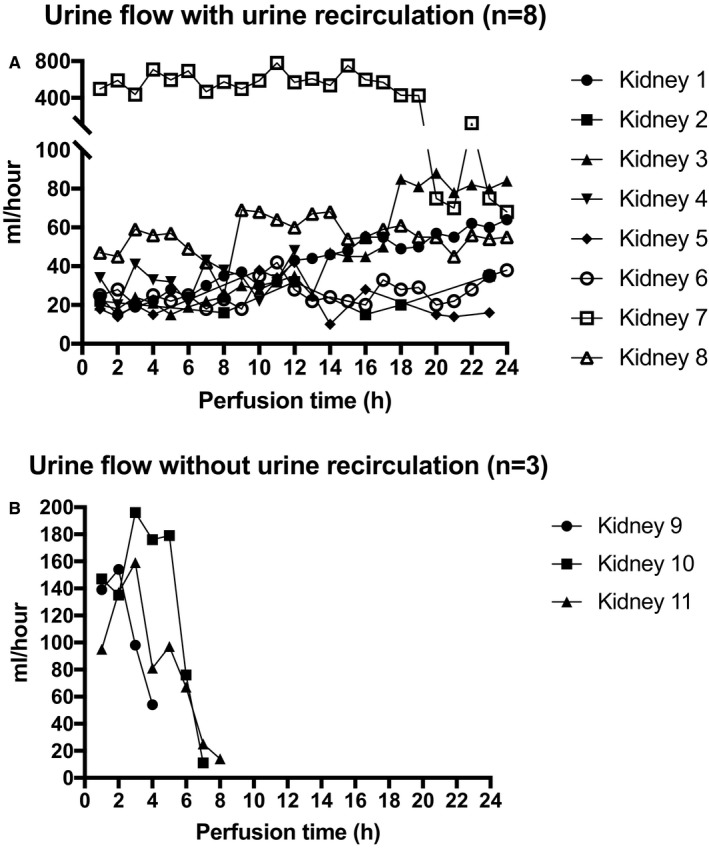
A. Urine flow: Hourly amounts per kidney during 24 hours of normothermic perfusion. B. Urine flow: Hourly amounts per kidney without urine recirculation during normothermic perfusion

Urine sodium levels of all except 2 kidneys, with and without urine recirculation, were low with sodium <100 mmol/L. In kidneys 4 (histologically proven cortical necrosis) and 7 (polyuric during perfusion, histologically proven acute‐on‐chronic hypertensive nephropathy), the urine sodium levels were the same as the perfusate values, 142 ± 3.9 mmol/L, potentially indicating tubular injury with lack of sodium reabsorption capacity. Hourly amounts of urine are displayed in Figure 5A,B for each kidney.

### 3.3 Biomarker measurements: NGAL and KIM‐1

Perfusate samples of the perfused kidneys were analyzed. The concentrations of all biomarkers increased during 24 hours of NMP from the first time point to the last time point of measurement in the urine recirculation group. This could be detected in all 9 analyzed kidneys (Table 5) and reflects a constant release of biomarkers into the perfusate during the process of normothermic preservation in a fully closed circuit with urine recirculation. To show differences between the kidneys, the delta concentrations of all biomarkers were calculated (value of last measurement minus value of first measurement) and the different profiles are shown in Figure S5A,B. The perfusate levels of both NGAL and KIM‐1 decreased over time in the group without urine recirculation, kidneys 9‐11. The exact values are shown in Table 5 and the different biomarker profiles are displayed in the Figure S5C,D. KIM‐1 could not be detected at all in kidney 9. It is worth noting that the absolute biomarker levels across the 2 groups without and with urine recirculation should not be directly compared, due to the replenishment of excreted urine with Ringer’s lactate solution in 1 group.

**Table 5:** Observed perfusate biomarker concentrations

	First time point^a^	Last time point^b^	Δ
NGAL, ng/mL with urine recirculation			
Kidney 1	12.627	28.159	15.532
Kidney 2	41.773	72.426	30.653
Kidney 3	34.470	112.231	77.762
Kidney 4	53.984	264.146	210.162
Kidney 5	18.418	105.254	86.836
Kidney 6	32.026	63.066	31.040
Kidney 7	59.108	259.944	200.836
Kidney 8	10.414	61.077	50.663
NGAL, ng/mL without urine recirculation			
Kidney 9	17.272	7.444	–9.828
Kidney 10	88.464	4.082	–84.382
Kidney 11	16.238	0.590	–15.648
KIM‐1, ng/mL with urine recirculation			
Kidney 1	0.727	3.033	2.305
Kidney 2	0.941	4.971	4.029
Kidney 3	1.319	1.834	0.515
Kidney 4	1.235	2.744	1.509
Kidney 5	0.503	3.085	2.582
Kidney 6	1.110	2.146	1.036
Kidney 7	0.079	0.583	0.504
Kidney 8	<Detection limit^c^	3.321	3.321
KIM‐1, ng/mL without urine recirculation			
Kidney 9	<Detection limit	<Detection limit	<Detection limit
Kidney 10	0.207	0.501	0.294
Kidney 11	0.645	0.533	–0.112

^a^One hour after perfusion start, ^b^after 24 hours of perfusion (kidneys 1‐8), after hours 6, 9, and 8 for kidneys 9‐11.

^c^Minimal detectable dose for assay ranged from 0.003 to 0.046 ng/mL.

### 3.4 Histology results

**Figure 6:**
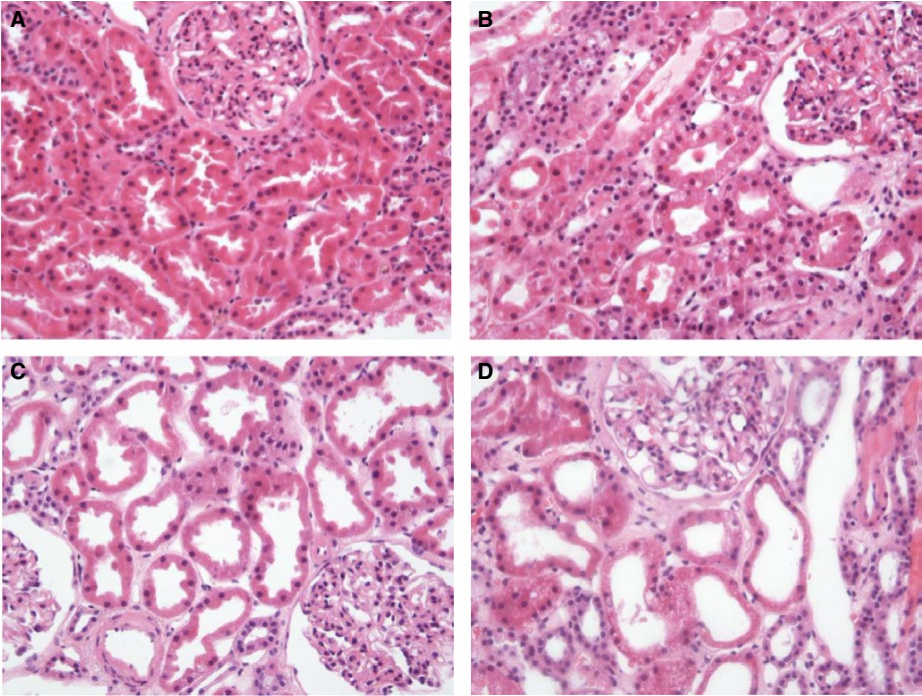
Histology photographs. A. Zero biopsy kidney 8. B. Biopsy after 24 hours of normothermic perfusion kidney 8. C. Zero biopsy kidney 11. D. Biopsy after 8 hours of normothermic perfusion kidney 11 [Color figure can be viewed at wileyonlinelibrary.com]

Tissue samples were obtained from all kidneys after 24 hours/at the end of perfusion. Acute tubular injury, of different severity grades, was seen in 10 kidneys (76.9%) preperfusion. The baseline tubular condition appeared unchanged after normothermic perfusion in 10 (76.9%) kidneys. Kidneys 1 and 6 showed improvement, and kidney 2 showed deterioration of baseline acute tubular injury, from a score of 0 to 1 [Correction added on June 22, 2018, after first online publication: “Kidneys 1 and 8” corrected to “Kidneys 1 and 6”]. In this kidney, widespread tubular epithelium vacuolation was observed in the 24‐hour sample. Vacuolation is a nonspecific finding that might be attributed to osmotically active perfusate compounds, such as mannitol (this might be the cause in our case), and to sustained ischemic damage. In this particular case, KIM‐1 immunoexpression was observed in 20% of the tubules on the 0‐hour biopsy and decreased to <5% positivity on the 24‐hour biopsy. Examples of histology results (before perfusion start and at the end of perfusion) for 1 kidney with urine recirculation (kidney 8) and for 1 kidney without urine recirculation (kidney 11) are illustrated in Figure 6. The photographs of hematoxylin and eosin staining for all other kidneys are shown in Figure S6. The tubular conditions and KIM‐1 immunohistochemistry results for all kidneys are displayed in Table 6.

**Table 6:** Histology results and KIM‐1 immunohistochemistry, tubular condition

Kidney	Tubular condition, baseline	Tubular condition, end of perfusion	KIM‐1 staining, baseline	KIM‐1 staining, end of perfusion
1	2	0	Positive, 5%	Positive, <5%
2	0	1 (Vacuolation)	Positive, 20%	Positive, <5%
3	0	0	Negative	Negative
4	3	3	Positive, 20%	Positive, <5%
5	0	0	Positive, 20%	Positive, <5%
6	2	1	Positive, <5%	Positive, <5%
7	2	2	Positive, 20%	Positive, 10‐5%
8	1	1	Positive, 5%	Positive, 5%
9	1	1	Negative	Negative
10	1	1	Negative	Negative
11	1	1	Positive, 5%	Positive, 5%

Presence of acute tubular injury: 0, absent; 1, loss of brush borders/vacuolation of tubular epithelial cells; 2 , cell detachment/cellular casts; 3, coagulation necrosis.

## 4 DISCUSSION

This is the first report of prolonged normothermic human kidney preservation and of the use of a closed perfusion circuit with urine recirculation. The prototype device was clearly capable of maintaining perfusion and enabled stabilization of the condition of a discarded human kidney for 24 hours. Sufficient oxygenation was delivered by incorporating 1 unit of RBCs (~250 mL) in the perfusate. The design of the device allowed real‐time monitoring and rapid alteration in the composition of oxygen and air delivered to the perfusate. This was particularly important as an isolated kidney requires very low levels of gas flow. Failure to achieve physiological po_2_, pco_2_, and pH would have had substantial metabolic effects over the course of 24 hours.

Different approaches to gas delivery have been used by other investigators. Brasile et al used 100% oxygen to maintain a partial pressure of O_2_ between 100 and 250 mm Hg (13.5‐34 kPa) in an acellular subnormothermic (32ºC) perfusion system (Exsanguinous Metabolic Support, Breonics, Watervliet, NY).^23^, ^24^ The regulation of pH and CO_2_ levels of the perfusate was achieved by intermittent gassing of the perfusion solution with CO_2_. The Selzner group used formulation combination of 95% O_2_ and 5% CO_2_ for oxygenating porcine kidneys in normothermic ex vivo kidney perfusion studies.^7^ Nicholson et al also described the use of 95% O_2_ and 5% CO_2._ 5

The practice of recycling urine into the perfusate has never been described in a normothermic perfusion device. One of the important outcomes of the current study is the demonstration of physiological stability over 24 hours. Urine recirculation is a novel feature of the circuit used in these experiments. We performed a hypothetical calculation to demonstrate that the lack of excretory function as a result of urine recirculation is most unlikely to have any significant adverse physiological consequences within the clinically relevant time frame (ie, several days). A 75‐kg human with 5 L of circulating volume in a stable state of renal function will develop clinical symptoms of renal failure after an anephric period of 3 days (as demonstrated by the interval between hemodialysis sessions). A single kidney has an approximate mass of 150‐180 g with a circulating volume of 500 mL: at a 500th of the mass and one‐tenth of the volume, renal replacement should be required 50 times less frequently than for an anephric patient, on the order of once every 150 days.

Three of our consecutive normothermic kidney perfusions were with volume replenishment (using Ringer’s lactate), as performed by previous investigators,^17^, ^18^ to provide a “standard” comparison to our urine recirculation group. In these kidney perfusions, it was not possible to maintain a physiological pH after 4‐6 hours of perfusion. The most significant difference was the higher sodium perfusate levels in the group without urine recirculation. Despite the small sample size, urine recirculation seems to be an optimal tool to keep a physiological perfusate composition for long‐term normothermic kidney perfusion.

In 2013, Nicholson et al published the first clinical study of renal transplant after ex vivo normothermic perfusion.^5^ Eighteen kidneys from extended criteria donors were perfused for ~1 hour at 35°C. The total amount of urine produced during 60 minutes of warm perfusion was between 50 and 450 mL. For short perfusion durations of this sort, the urine output can be replenished easily and in an uncomplicated way with Ringer’s lactate: this was demonstrated by Hosgood and Nicholson in several publications.^9^, ^10^, ^11^, ^12^ However, for longer‐term perfusions, the disruption of perfusate electrolyte content and pH is potentially problematic. Further investigations will be required to determine whether a physiological perfusate sodium level or other urine metabolites are essential for maintenance of acid‐base balance.

Compared with results from normothermic liver perfusion,^25^ lactate did not seem to correlate with a poor or good performance of the kidney during NMP—not in the urine recirculation group or in the group with urine replenishment. According to the literature, it is most likely that lactate levels are related with active glucose metabolism in the kidney—both glycolysis and gluconeogenesis.^26^, ^27^, ^28^, ^29^ The lactate metabolism of the native human kidney plays a major role and the renal cortex appears to be the most important lactate‐consuming organ after the liver.^26^ Bartlett et al showed years ago that the renal function affects lactate and glucose metabolism.^27^ The kidney contributes to glucose homeostasis via different pathways: gluconeogenesis, glucose filtration, glucose reabsorption, and glucose consumption.^28^ There seems to be a corticomedullary glucose–lactate recycling system. The medulla consumes glucose via active glycolysis and generates lactate. The cortex has the ability to take up the lactate released by the medulla and uses it for oxidation and gluconeogenesis.^27^ Furthermore, Bartlett et al demonstrated in their early publication that lactate production also correlates with the urine flow rate and sodium resorption. However, they stated that lactate consumption did not correlate with renal function.^27^ Although the numbers in our study are too low to demonstrate a potential correlation with urine flow rate and sodium resorption, we also could not see any correlation between perfusion parameters and histology results.

Interpretation of the findings of this small cohort of 24‐hour–perfused kidneys is limited by the fact that none of the organs were transplanted; it therefore was not possible to corroborate the data we collected with transplant function. However, the progressive increase in the levels of NGAL and KIM‐1 in the perfusate does align with the biomarker analysis in the clinical trial (investigating HMP) by Parikh et al, which demonstrated an increase in all biomarkers during perfusion.^30^ Although a kidney on an HMP device is not producing any urine, our NMP prototype and the HMP device can be compared as both tools demonstrate a closed perfusion circuit with the same perfusate throughout. The interpretation and the informative value of detected biomarkers might be more difficult compared with kidney perfusion setups where the urine is excreted and replaced. We have shown during perfusion without urine recirculation that the NGAL and KIM‐1 levels clearly decreased over time. However, due to the small number of perfused kidneys, predictive value of these markers will need to be assessed in a larger cohort. Hosgood and Nicholson analyzed the values of urine NGAL and KIM‐1 after short‐term normothermic kidney perfusion. Higher levels of urine NGAL, but not KIM‐1, were associated with a raised donor creatinine before organ retrieval.^31^ Interestingly, in our study, kidney 4, which appeared to be the one with the most significant ischemic injury, lowest urine output, and cortical necrosis on biopsy, had the greatest increase in NGAL. In the Hosgood analysis, KIM‐1 levels were not associated with perfusion parameters or renal function in the donor.^25^ An increase of KIM‐1 in a closed perfusion circuit could be of interest in future, but the numbers in the present study are insufficient to draw robust conclusions. Alongside its potential role as an early biomarker of acute tubular injury,^32^ it has been suggested that the shedding of KIM‐1 in the kidney undergoing regeneration is an active process that allows dedifferentiated tubular cells to scatter on denuded patches of the basement membrane and reestablish a continuous epithelial layer.^33^ Histologically, the presence of donor lesions and baseline conditions (interstitial fibrosis and tubular atrophy, arterial fibroelastosis, or arteriolar hyalinosis) was not associated with the development of acute tubular injury after NMP. In all except 1 kidney, the baseline tubular condition was preserved or improved. Only in kidney 2, a poorly perfused DBD kidney from an elderly donor, did the tubular condition deteriorate (vacuolation).

We have demonstrated, for the first time, that NMP of the human kidney is feasible for 24 hours and that the recycling of urine is an effective method of maintaining perfusate homeostasis and acid‐base stability. The preliminary data on perfusate biomarkers are encouraging and require corroboration with posttransplant data in due course. The ability to maintain (and possibly improve) the condition of donor kidneys of marginal quality for long enough to carry out viability assessment could increase the feasibility to exploit this important source of donor organs.

## ACKNOWLEDGMENTS

The authors gratefully acknowledge funding and support from the UK’s National Institute for Health Research under an Invention for Innovation (i4i) award (II‐ES‐1010‐10096) and from the Engineering and Physical Sciences Research Council under the Oxford Centre for Drug Delivery Devices (OxCD3) programme grant (EP/L024012/1).

## DISCLOSURE

The authors of this manuscript have conflicts of interest to disclose as described by the *American Journal of Transplantation*. In addition to being full‐time academics at the University of Oxford, PJF and CCC receive consultancy payments as nonexecutive medical and technical directors of OrganOx Limited, and are shareholders. The other authors have no conflicts of interest to disclose.
