# Supplementary material for: Twenty‐four–hour normothermic perfusion of discarded human kidneys with urine recirculation
Source: Am J Transplant. 2018 Jun 20;19(1):178–92. doi: 10.1111/ajt.14932 (PMC6491986; doi:10.1111/ajt.14932)
Supplement: Supplementary file 1 [file AJT-19-178-s001.pdf]

Appendix: Supplementary Figures

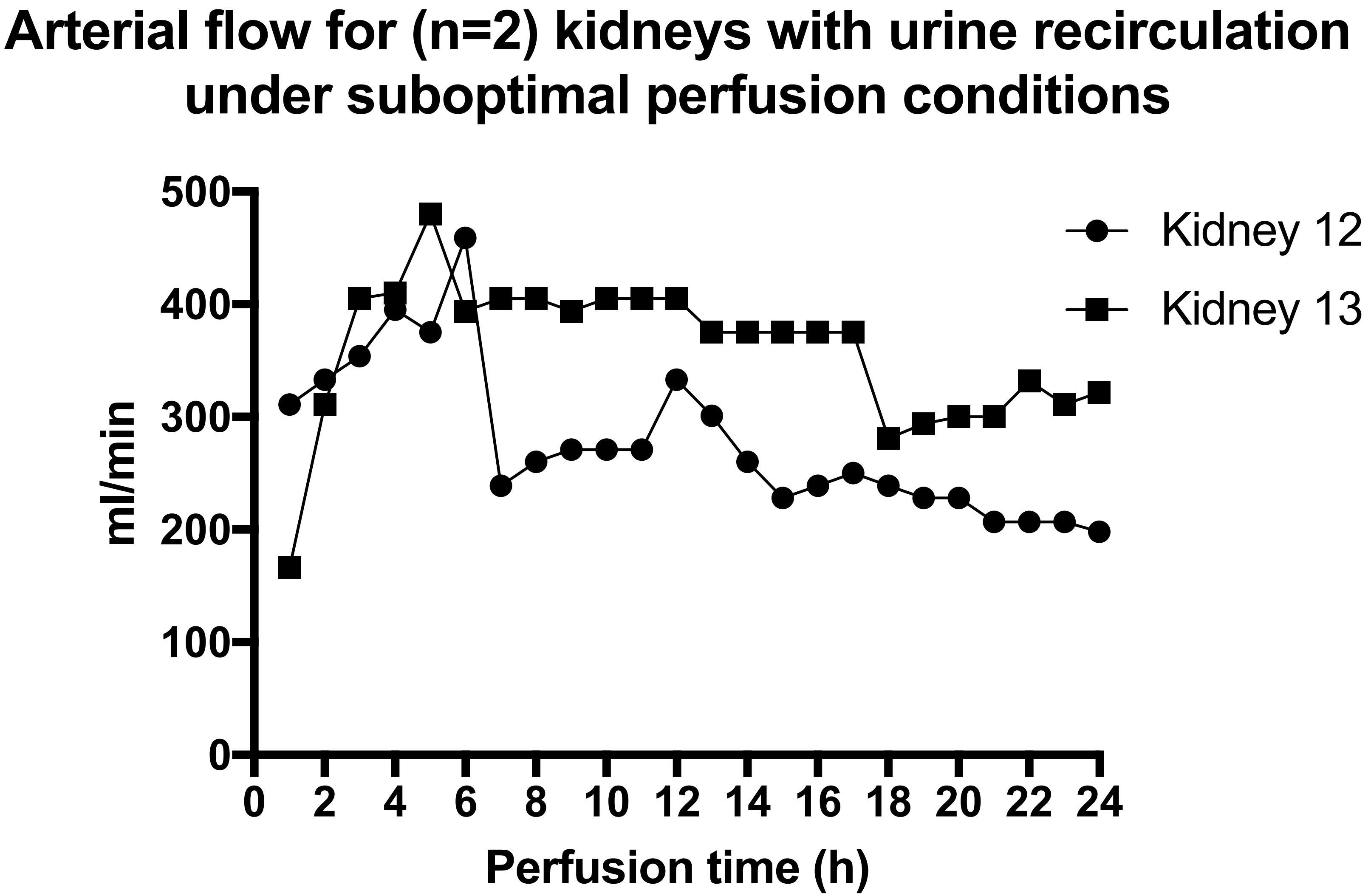

Supplementary Figure 1

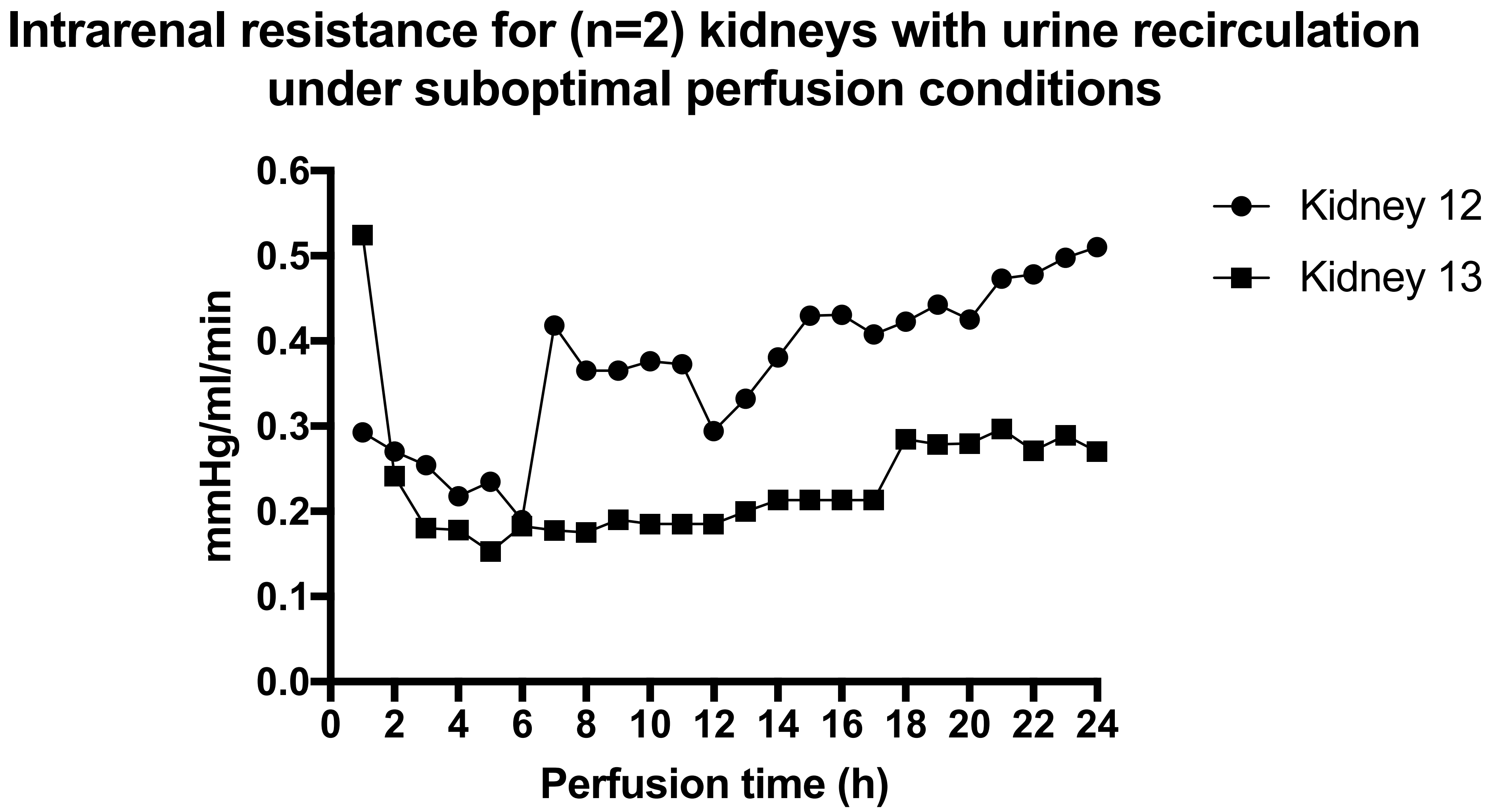

Supplementary Figure 2

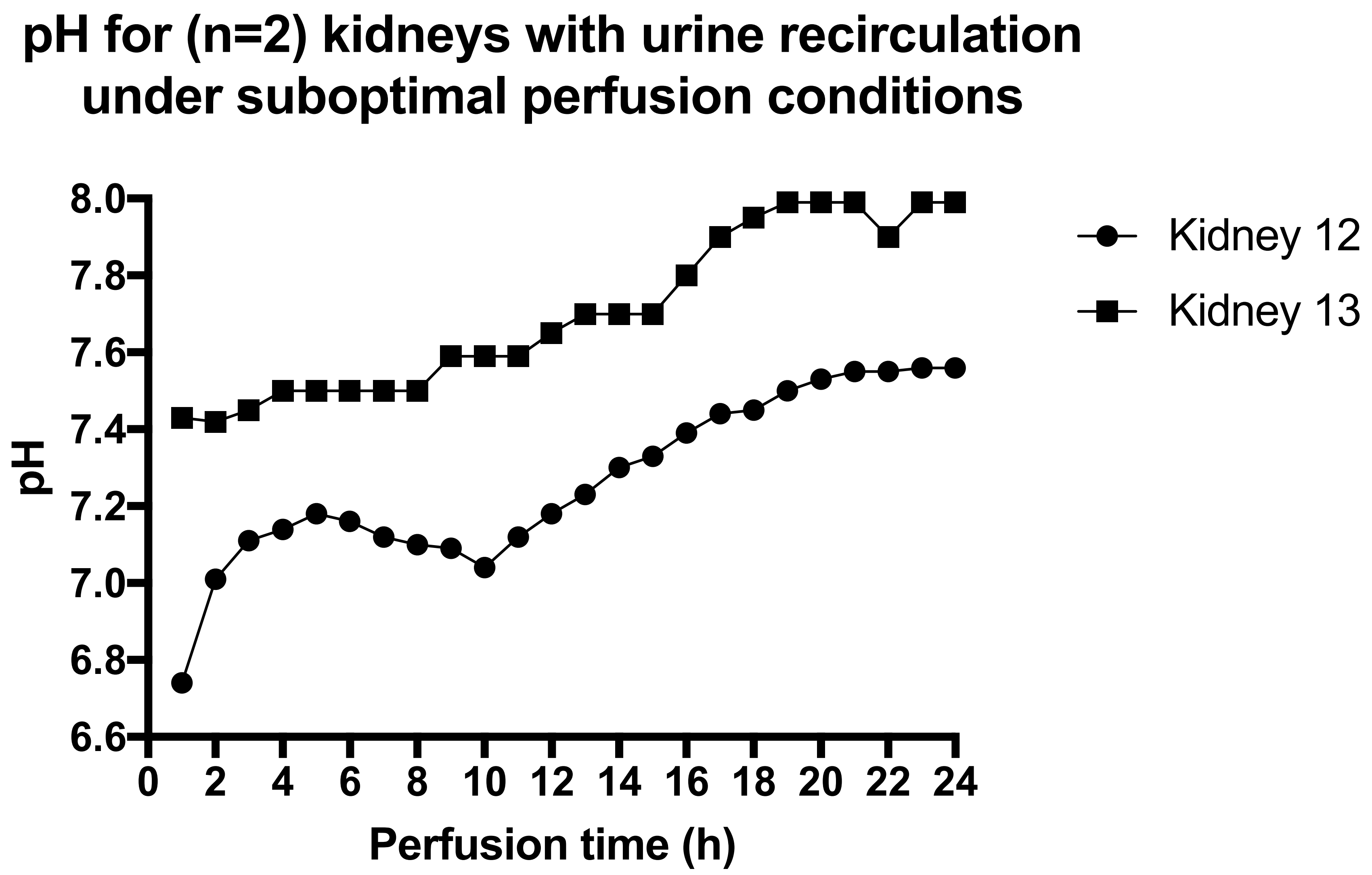

Supplementary Figure 3

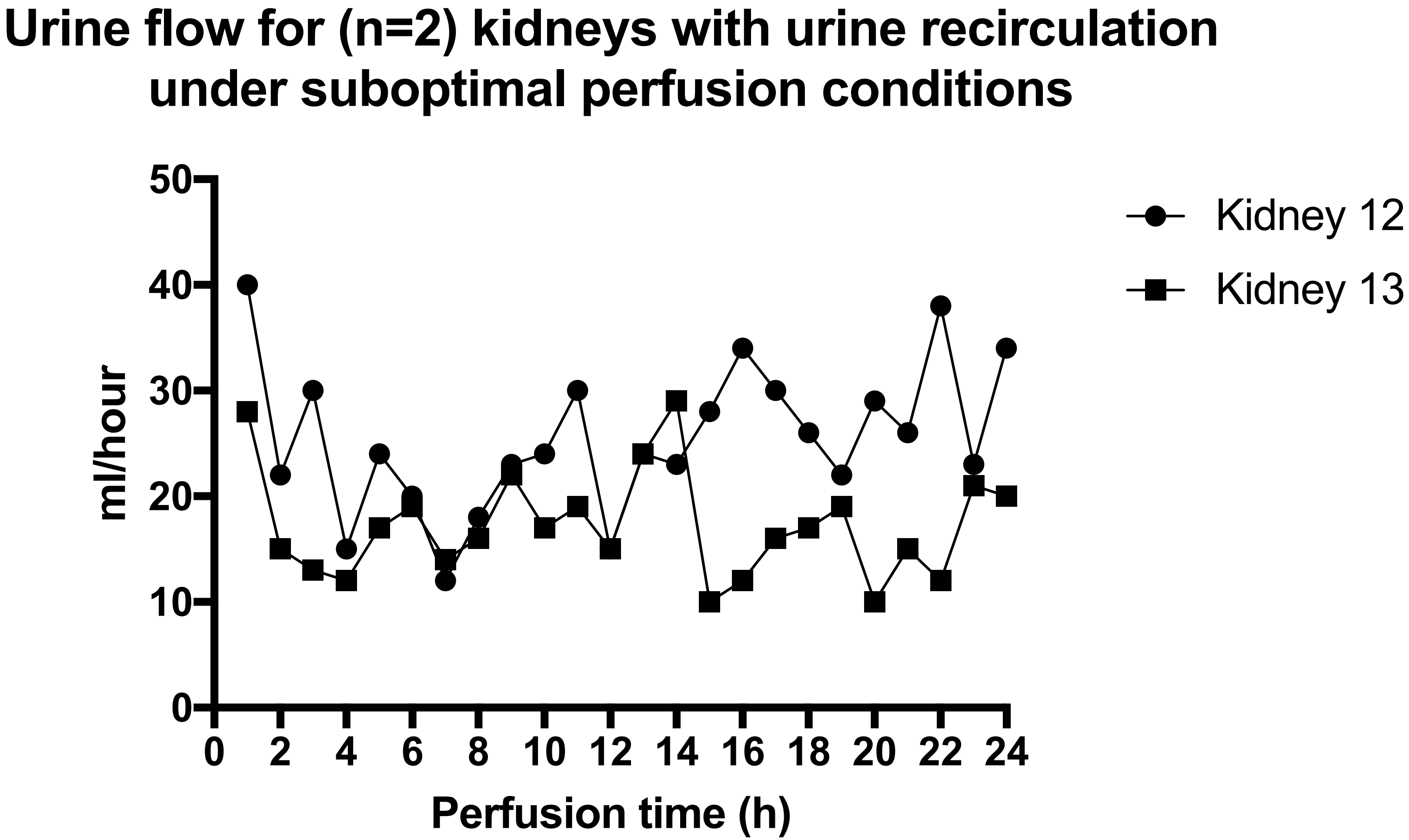

Supplementary Figure 4

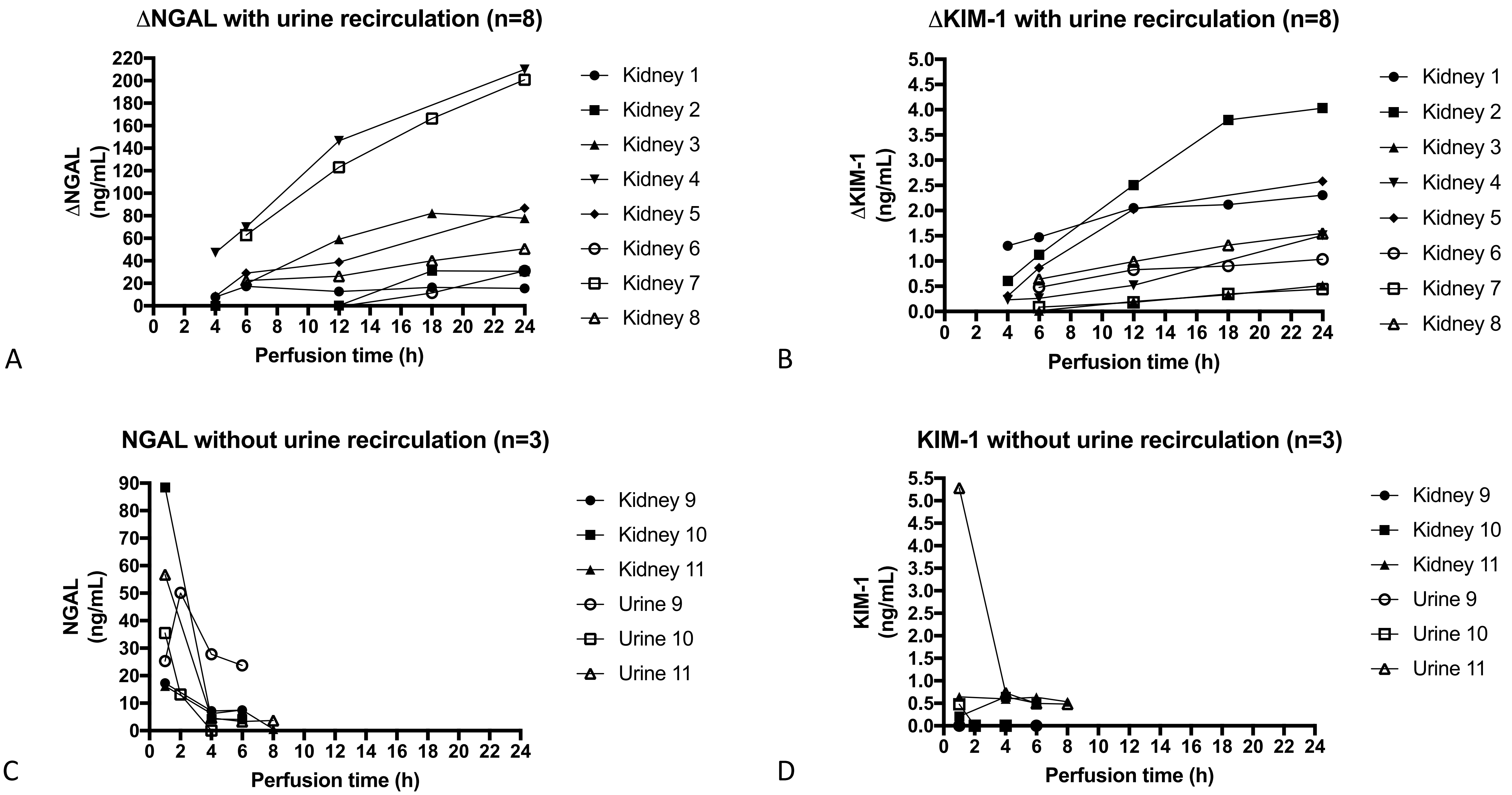

Supplementary Figure 5

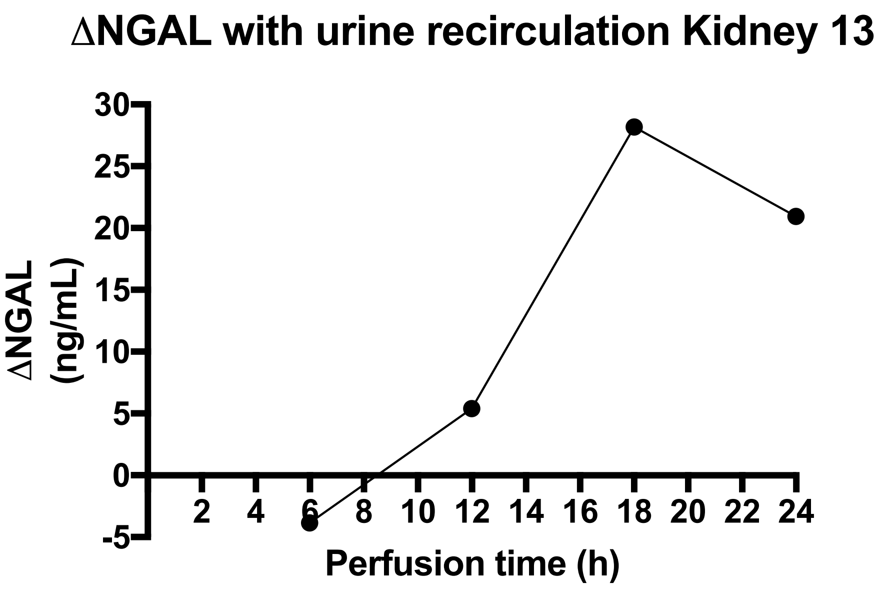

E

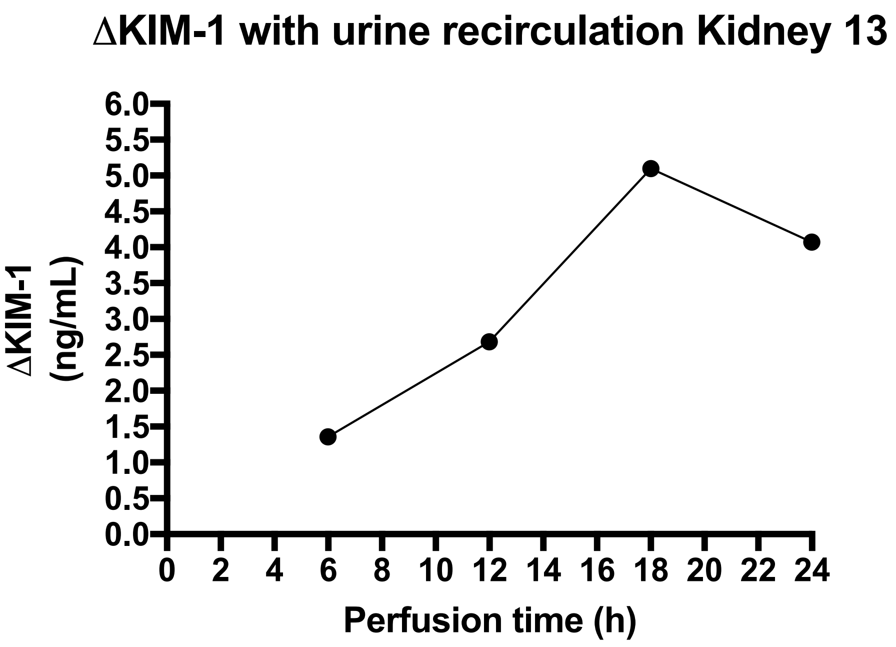

F

Supplementary Figure 5

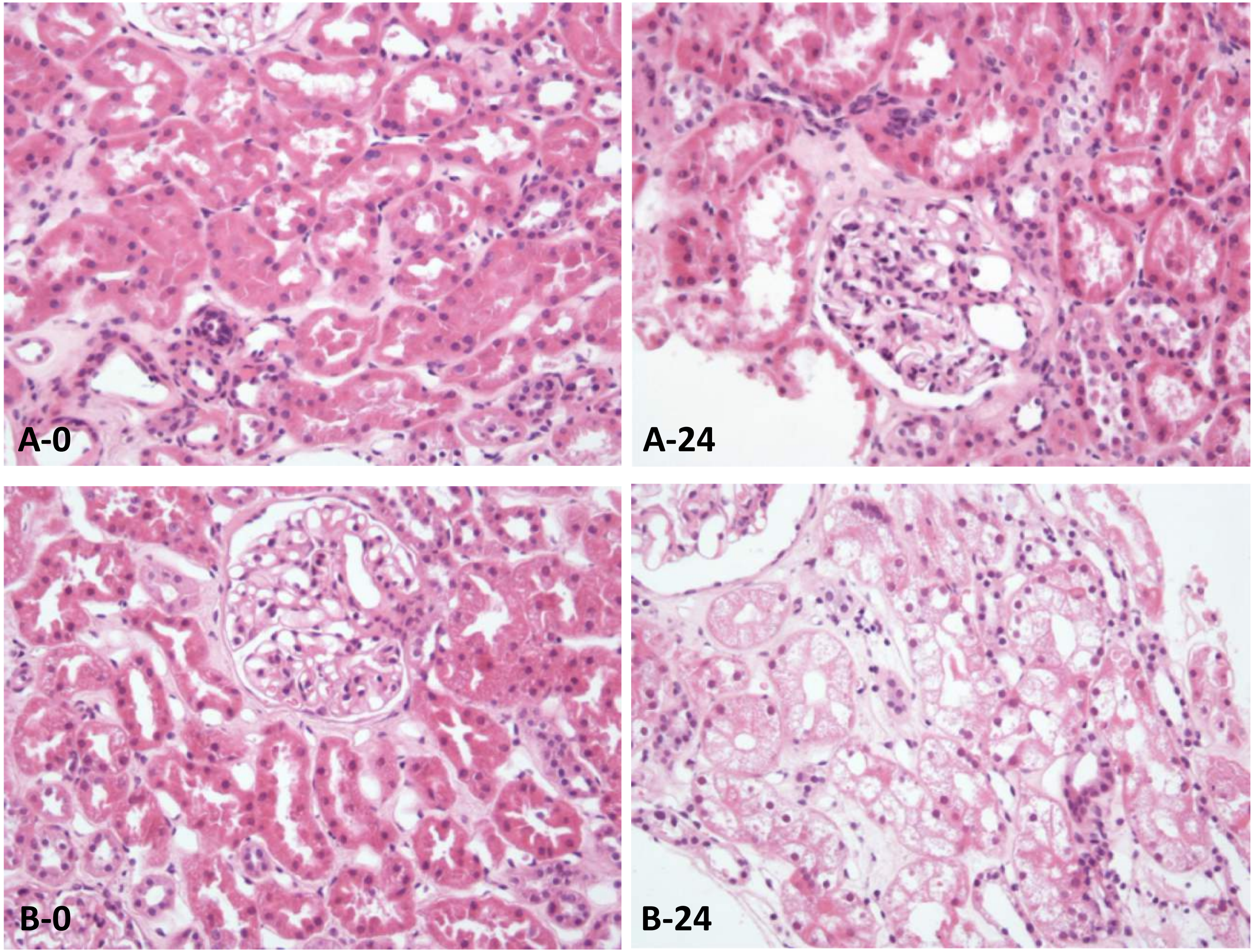

Supplementary Figure 6

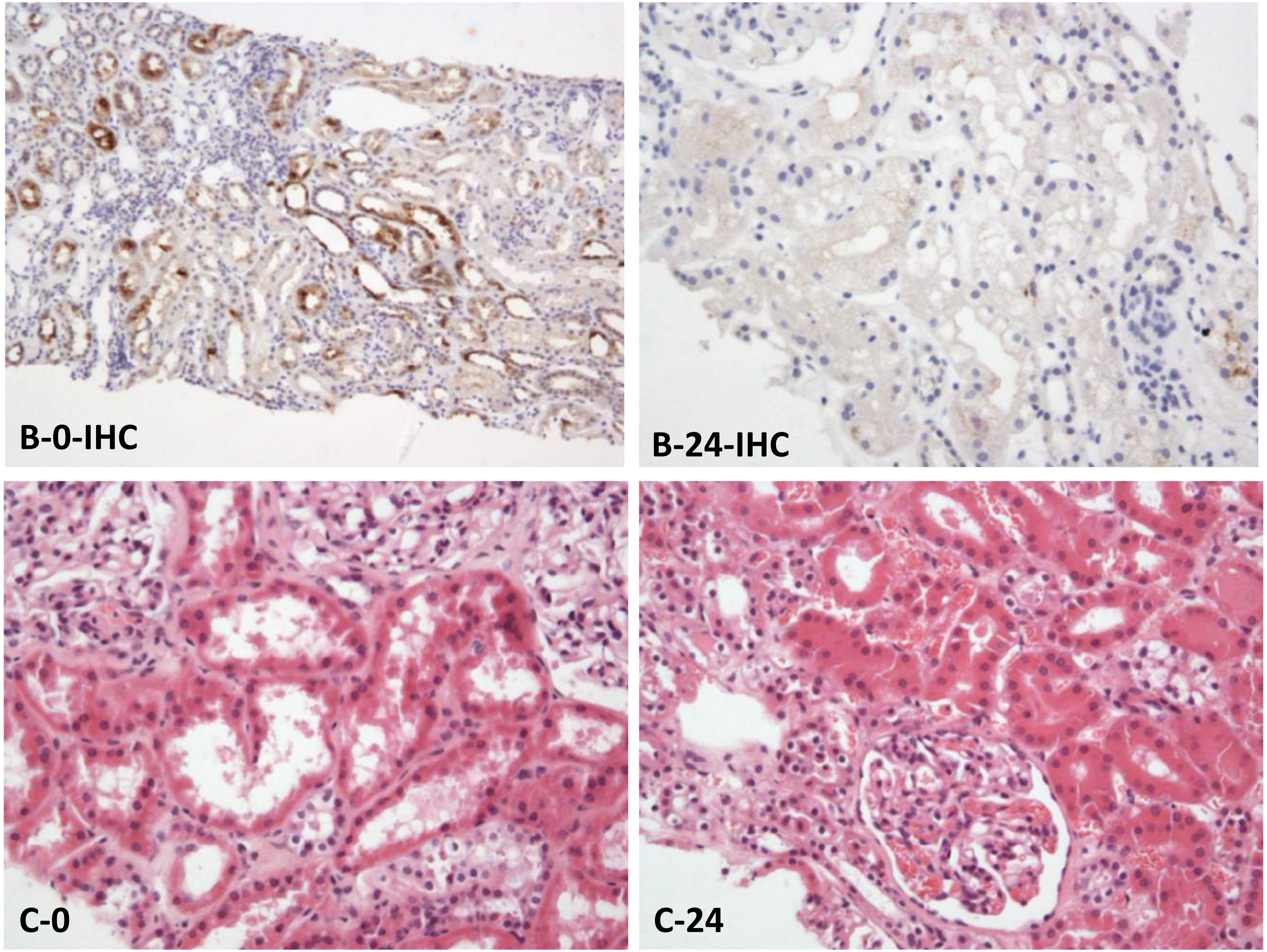

Supplementary Figure 6

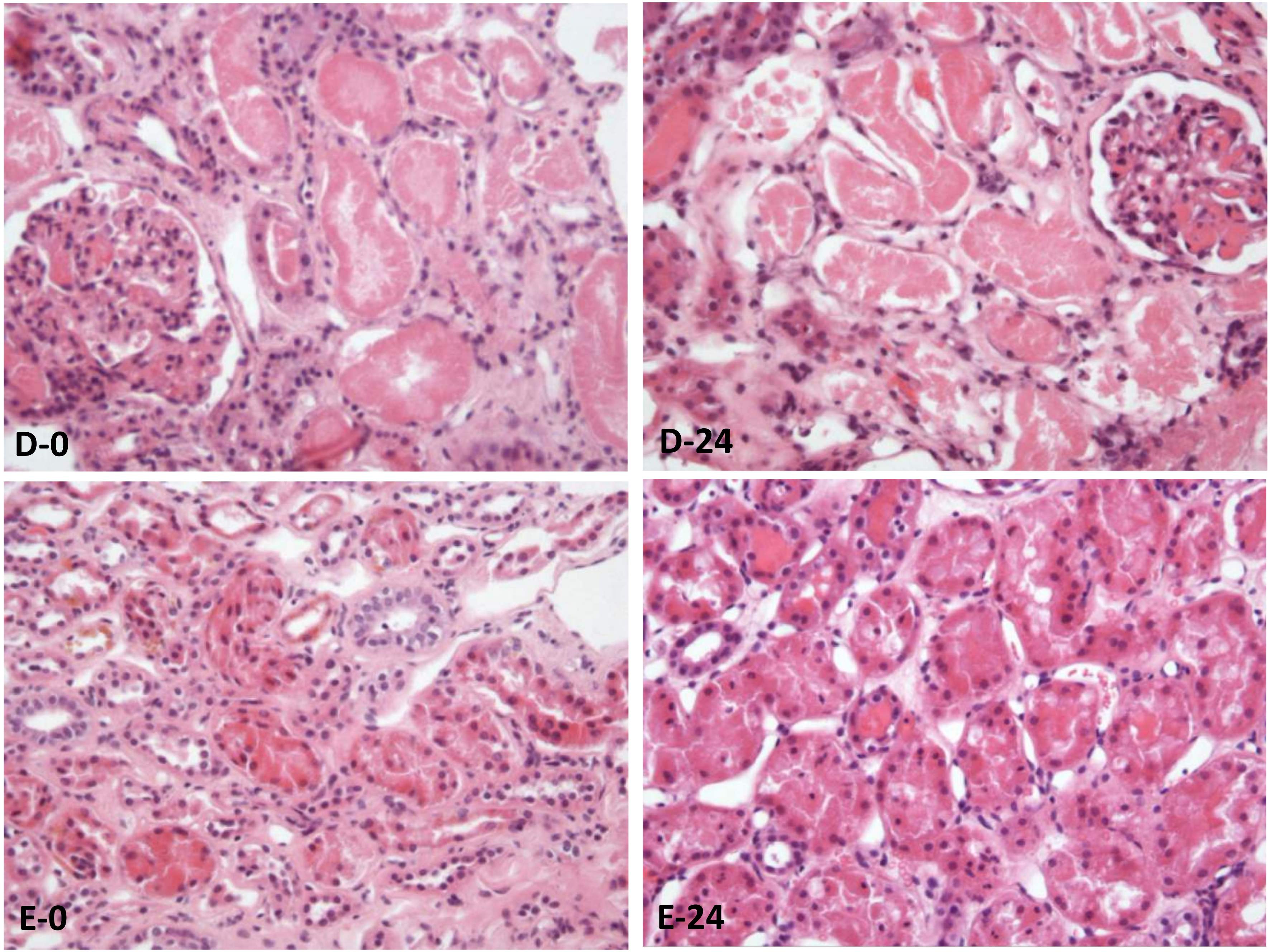

Supplementary Figure 6

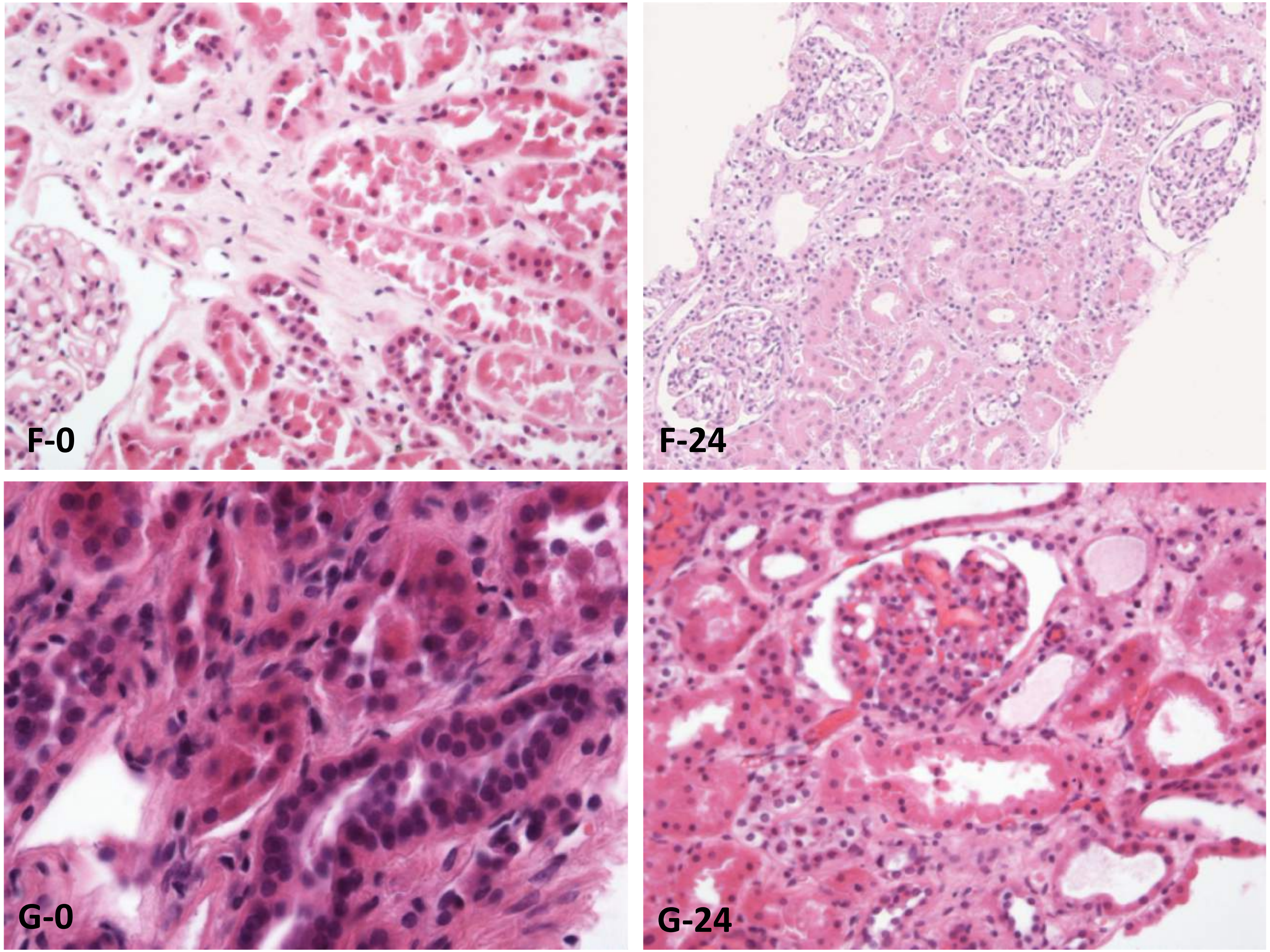

Supplementary Figure 6

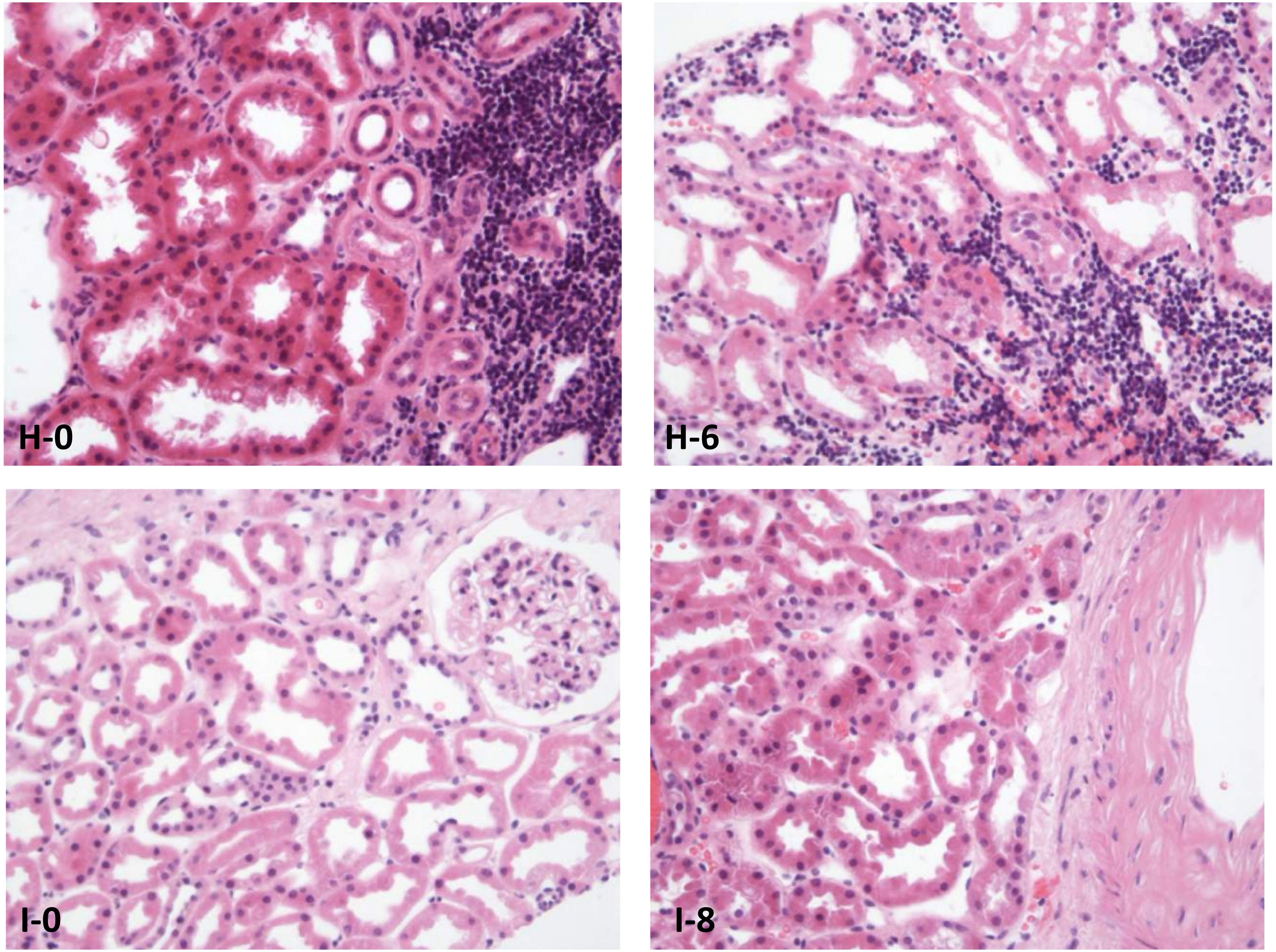

Supplementary Figure 6

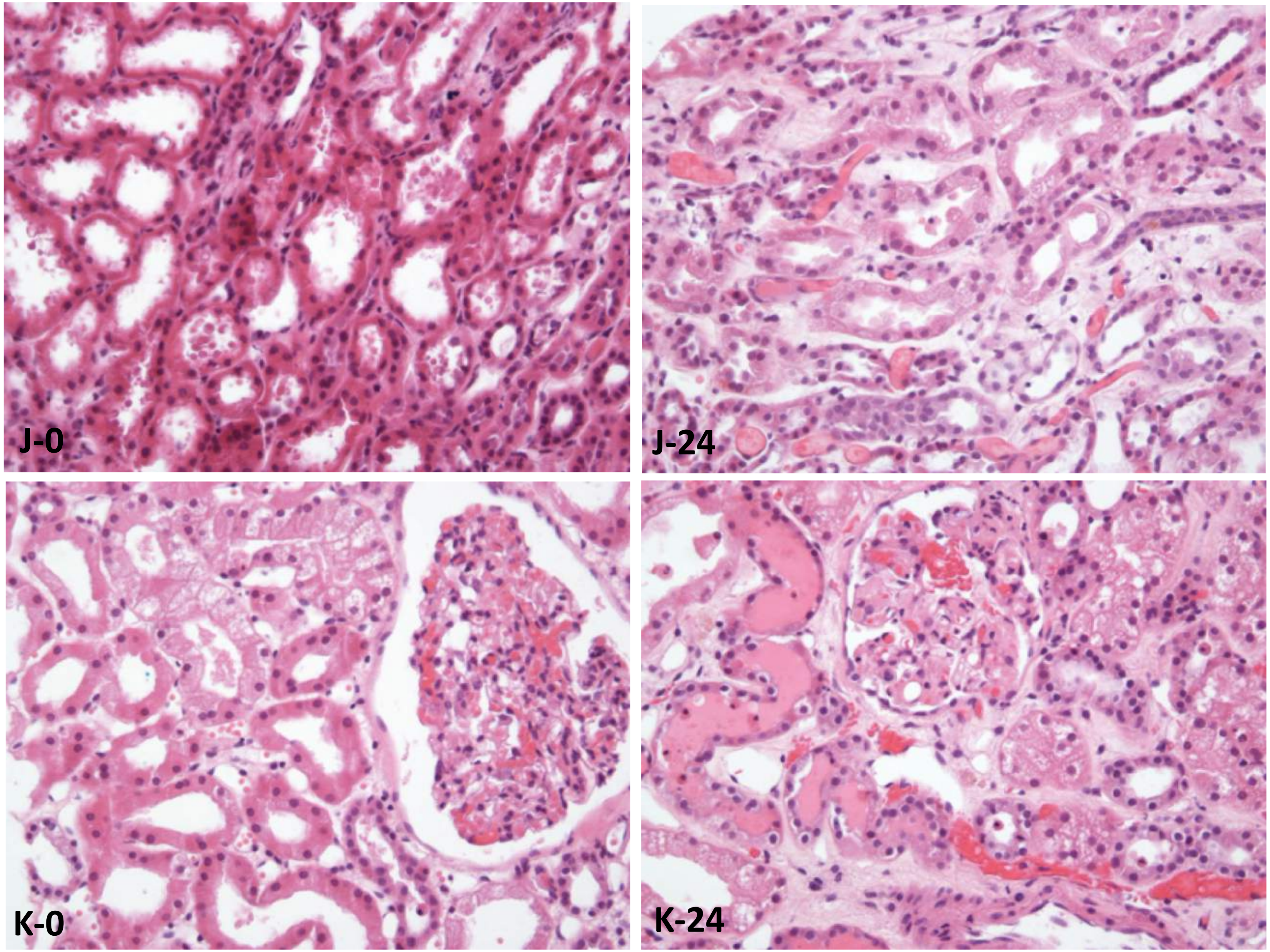

Supplementary Figure 6
