# Supplementary material for: Twenty‐four–hour normothermic perfusion of discarded human kidneys with urine recirculation
Source: Am J Transplant. 2018 Jun 20;19(1):178–92. doi: 10.1111/ajt.14932 (PMC6491986; doi:10.1111/ajt.14932)
Supplement: Supplementary file 2 [file AJT-19-178-s002.docx]

| **Supplementary Table 1: Organ procurement parameters and reasons for discard of kidneys 12 and 13** | | | | | | | |  |
| --- | --- | --- | --- | --- | --- | --- | --- | --- |
|  | Age in years | Sex | BMI in kg/m2 | Donor Type | WIT in min | CIT in hours+min | Hypertension | Reason for Discard |
| Kidney 12 | 67 | female | 27.9 | DBD | n.a. | 18 + 31 | no | patchy perfusion |
| Kidney 13 | 38 | male | 20.9 | DCD | 10 | 29 + 9 | yes | 50% glomerulosclerosis inflammatory changes |

| **Supplementary Table 2: Donor characteristics of kidney 12 and 13** | **Kidney 12** | **Kidney 13** |
| --- | --- | --- |
|  |  |  |
| Donor age in years | 68 (47-78) | 50 (38-74) |
| Donor BMI kg/m2 | 29.2 ± 6.1 | 23.8 ± 2.2 |
|  |  |  |
| Serum crea at admission in µmol/l | 96.1 ± 38.2 | 82 ± 26.7 |
| Serum crea at retrieval in µmol/l | 86 ± 37 | 67.2 ± 46.1 |
| Serum urea at admisison in mmol/l | 6.1 ± 2.3 | 4.8 ± 1.4 |
| Serum urea at retrieval in mmol/l | 6.6 ± 3 | 7.7 ± 6.5 |
| Urine output last hour in ml | 147.1 ± 138 | 110.8 ± 76.2 |
| Urine output last 24 hours in ml | 3500 ± 1277 | 4215 ± 3357 |
|  |  |  |
| CIT in hours | 30.9 ± 34.1 | 35 ± 34.35 |
| WIT in minutes | n.a. | 15.5 ± 9.1 |
|  |  |  |
| DBD = donation after brain death |  |  |
| DCD = donation after circulatory death |  |  |
| CVA = cerebro vascular accident |  |  |
| CIT = cold ischemia time |  |  |
| WIT = warm ischemia time |  |  |
| n.a. = not applicable |  |  |

| **Supplementary Table 3: Hemodynamic and metabolic function parameters for kidney 12 and 13** | **Kidney 12** | **Kidney 13** |
| --- | --- | --- |
|  |  |  |
| Arterial pressure in mmHg (mean, SD) | 97±5.3 | 78.9±6.2 |
| Arterial flow in ml/min (mean, SD) | 277.7±67.3 | 357.1±65.7 |
| IRR in ml/min/mmHg (mean, SD) | 0.37±0.09 | 0.24±0.08 |
| pH (mean, SD) | 7.26±0.22 | 7.72±0.23 |
|  |  |  |
| arterial pO2 in kPa (mean, SD)/ in mmHg (mean, SD) | 11.4±5.6/ 85.7±42.1 | 14.6±2.5/ 109.8±18.8 |
| venous pO2 in kPa (mean, SD)/ in mmHg (mean, SD) | 7.3±1/ 54.9±7.5 | 7.8±1.7/ 58.6±12.8 |
| arterial pCO2 in kPa (mean, SD)/ in mmHg (mean, SD) | 4±2/ 30±15 | 1.1±0.5/ 8.3±3.8 |
|  |  |  |
| Lactate level in mmol/l (mean, SD) | not available | 11.52±5.8 |
| Total glucose given in mg* | 12 | 0 |
| Total urine output in ml/24 hours | 412 | 630 |
| Urine recirculation yes/no | no | no |
| Time on the device (hours+min) | 24 | 24+25 |
|  |  |  |
| *circulating perfusate volume of 500ml |  |  |

| **supplementary Table 4: Observed perfusate biomarker concentrations** | **First Time Point*** | **Last Time Point**** | **Delta** |
| --- | --- | --- | --- |
| **NGAL in ng/mL with urine recirculation** | |  |  |
| Kidney 13 | 28.429 | 49.361 | 20.932 |
|  |  |  |  |
| **KIM-1 in ng/mL with urine recirculation** | |  |  |
| Kidney 13 | 0.895 | 4.967 | 4.072 |
|  |  |  |  |
| * 1 hour after perfusion start, ** after 24 hours of perfusion |  |  |  |
| *** there were no perfusate samples available for kidney 12 due to logistical reasons. | | |  |

| **supplementary Table 5: Histology results and KIM-1 immunohistochemistry - tubular condition of kidneys 12 and 13** | | | | |  |  |  |
| --- | --- | --- | --- | --- | --- | --- | --- |
| Kidney | Baseline tubular condition | Tubular condition end of perfusion | KIM-1 staining baseline | KIM-1 staining end of perfusion |  |  |  |
| 12 | 1 | 1 | positive, 20% | positive, 20% |  |  |  |
| 13 | 1 | 1 | positive, 30% | positive, 20% |  |  |  |
|  |  |  |  |  |  |  |  |
| Presence of acute tubular injury: 0 – absent; 1 – loss of brush borders / vacuolation of tubular epithelial cells; 2 – cell detachment / cellular casts; 3 – coagulation necrosis | | | | | | | |
|  |  |  |  |  |  |  |  |
