# Supplementary material for: Twenty‐four–hour normothermic perfusion of discarded human kidneys with urine recirculation
Source: Am J Transplant. 2018 Jun 20;19(1):178–92. doi: 10.1111/ajt.14932 (PMC6491986; doi:10.1111/ajt.14932)
Supplement: Supplementary file 3 [file AJT-19-178-s003.docx]

**Supporting Information**

Additional Supporting Information may be found online in the supporting information tab for this article.

Table S1: Organ procurement parameters and reasons for discard of kidneys 12 and 13

Table S2: Donor characteristics kidneys 12 and 13

Table S3: Hemodynamic and metabolic function parameters for kidney 12 and 13

Table S4: Observed perfusate biomarker concentrations in kidney 13

Table S5: Histology results and KIM-1 immunohistochemistry - tubular condition of kidney 12 and 13

Figure S1: Arterial flow values in ml/min for kidneys 12 and 13

Figure S2: Intrarenal resistance values in mmHg/ml/min for kidneys 12 and 13 over time

Figure S3: pH values for kidneys 12 and 13 during 24 hours of normothermic perfusion

Figure S4: Urine flow: Hourly amounts during 24 hours of normothermic perfusion in kidneys 12 and 13

Figure S5: Biomarker profiles: Delta values of NGAL (A), KIM-1 (B) in kidney perfusates with urine recirculation. NGAL (C) and KIM-1 (D) values in kidney perfusates without urine recirculation. Delta values of NGAL (E) and KIM-1 (F) in perfusate of kidney 13.

Figure S6: Histology photographs

A-0: Zero biopsy kidney 1.

A-24: Biopsy after 24 hours of normothermic perfusion kidney 1.

B-0: Zero biopsy kidney 2.

B-24: Biopsy after 24 hours of normothermic perfusion kidney 2.

B-0-IHC: KIM-1 immunostaining in zero biopsy of kidney 2. B-24-IHC: KIM-1 immunostaining after 24 hours of normothermic perfusion in kidney 2.

C-0: Zero biopsy kidney 3.

C-24: Biopsy after 24 hours of normothermic perfusion kidney 3.

D-0: Zero biopsy kidney 4.

D-24: Biopsy after 24 hours of normothermic perfusion kidney 4.

E-0: Zero biopsy kidney 5.

E-24: Biopsy after 24 hours of normothermic perfusion kidney 5.

F-0: Zero biopsy kidney 6.

F-24: Biopsy after 24 hours of normothermic perfusion kidney 6.

G-0: Zero biopsy kidney 7.

G-24: Biopsy after 24 hours of normothermic perfusion kidney 7.

H-0: Zero biopsy kidney 9. Noticeable the monomorphic cell infiltrate.

H-6: Biopsy after 6 hours of normothermic perfusion kidney 9. Noticeable the monomorphic cell infiltrate.

I-0: Zero biopsy kidney 10.

I-8: Biopsy after 8 hours of normothermic perfusion kidney 10.

J-0: Zero biopsy kidney 12.

J-24: Biopsy after 24 hours of normothermic perfusion kidney 12.

K-0: Zero biopsy kidney 13.

K-24: Biopsy after 24 hours of normothermic perfusion kidney 13.
